# Facilitating the dry reforming of methane with interfacial synergistic catalysis in an Ir@CeO_2−*x*_ catalyst

**DOI:** 10.1038/s41467-024-48122-6

**Published:** 2024-05-04

**Authors:** Hui Wang, Guoqing Cui, Hao Lu, Zeyang Li, Lei Wang, Hao Meng, Jiong Li, Hong Yan, Yusen Yang, Min Wei

**Affiliations:** 1 State Key Laboratory of Chemical Resource Engineering, Beijing Advanced Innovation Center for Soft Matter Science and Engineering, Beijing University of Chemical Technology, 100029 Beijing, P. R. China; 2 State Key Laboratory of Heavy Oil Processing, China University of Petroleum (Beijing), 102249 Beijing, P. R. China; 3 Quzhou Institute for Innovation in Resource Chemical Engineering, 324000 Quzhou, P. R. China; 4 Shanghai Synchrotron Radiation Facility, Shanghai Institute of Applied Physics, Chinese Academy of Sciences, 201204 Shanghai, P. R. China

**Keywords:** Heterogeneous catalysis, Catalytic mechanisms

## Abstract

The dry reforming of methane provides an attractive route to convert greenhouse gases (CH_4_ and CO_2_) into valuable syngas, so as to resolve the carbon cycle and environmental issues. However, the development of high-performance catalysts remains a huge challenge. Herein, we report a 0.6% Ir/CeO_2−*x*_ catalyst with a metal-support interface structure which exhibits high CH_4_ (~72%) and CO_2_ (~82%) conversion and a CH_4_ reaction rate of ~973 μmol_CH4_ g_cat_^−1^ s^−1^ which is stable over 100 h at 700 °C. The performance of the catalyst is close to the state-of-the-art in this area of research. A combination of in situ spectroscopic characterization and theoretical calculations highlight the importance of the interfacial structure as an intrinsic active center to facilitate the CH_4_ dissociation (the rate-determining step) and the CH_2_* oxidation to CH_2_O* without coke formation, which accounts for the long-term stability. The catalyst in this work has a potential application prospect in the field of high-value utilization of carbon resources.

## Abstract (web summary)

Dry reforming of methane is an attractive route to convert greenhouse gases into valuable syngas. Here, the authors demonstrate design strategy to prepare efficient catalysts towards activation of both C–H and C = O bonds and provide atomic-level insights into interfacial synergistic catalysis.

## Introduction

Owing to the increasing global warming and climate change issues, strategies for greenhouse gas reduction have drawn extensive interest from both fundamental research and industrial applications^1–3^. CO_2_ and CH_4_ are regarded as two predominant contributors to the greenhouse effect; therefore, their utilization and conversion to high-value-added chemicals and fuels meet the demands for achieving large-scale carbon fixation, carbon emission reduction and carbon cycle^4–7^. One promising approach is to convert both CO_2_ and CH_4_ simultaneously through thermo-catalytic dry reforming of methane (DRM) reaction, which produces the syngas (H_2_ and CO) as an important platform for alternatives of petroleum-derived fuels and valuable chemicals^8–11^. Thermodynamically, the DRM reaction involves both C–H bond dissociation (439 kJ mol^−1^) and C=O bond hydrogenation (750 kJ mol^−1^) followed by subsequent formation of CO and H_2_, resulting in a highly endothermic process (Δ*H*_298K_ = 247 kJ mol^−1^)^12–15^. This normally requires a high energy consumption and rigorous reaction temperature (>800 °C) to maintain favorable catalytic activity, but suffers from serious catalyst deactivation due to nanoparticle agglomeration and carbon deposition^16–18^. In this case, great efforts have been focused on the exploration of catalysts towards DRM reaction, such as supported noble metals (e.g., Pt^19^, Ru^9,20^, and Pd^21,22^) and non-noble metals (e.g., Ni^23–25^ and Co^26^) catalysts. Although considerable advances have been made, rational design and preparation of highly efficient catalysts to acquire high activity and stability simultaneously, still remain a big challenge.

In general, pure metal surfaces exhibit low reactivity towards methane dissociation and are prone to deactivation resulting from carbon deposition; whilst both experimental and theoretical studies have shown that C–H bond activation is more sensitive to coordinatively unsaturated metallic sites^12,27^. In this respect, the emerging strong metal-support interaction (SMSI) has demonstrated many appealing advantages, such as the interfacial structure and synergistic catalysis, which have attached widespread research interest in various heterogeneous reactions (e.g., CO_2_ methanation and water gas shift reaction)^28–31^. The fine-tuning for SMSI has been successfully employed to optimize geometric/electronic structure of metal species at the interface^32–34^, which provides great opportunities to promote catalytic performance towards DRM reaction. On the one hand, the oxidic M^*δ+*^ metal species formed at the interfacial sites as an electron–acceptor, would reduce the *T*_d_ symmetry structure of methane molecule and thus facilitate its activation dehydrogenation to CH_*x*_^6,12,20^. For instance, Pirovano et al. reported that Ni^2+^ species promotes C−H bond dissociation at a lower temperature relative to metal Ni based on experiments and DFT calculations^35^. On the other hand, reducible supports (e.g., CeO_2_, ZrO_2_, and TiO_2_), which renders a facile conversion between two oxidation states (e.g., Ce^4+^ and Ce^3+^), would stabilize oxidic M^*δ+*^ species via accommodating metal-to-support electron transfer^32,36–38^. Meanwhile, the concomitant oxygen vacancies make a great contribution to elevate the activation adsorption of C=O group and facilitate the transformation of intermediates^34,39,40^. For example, Liu et al. reported the oxygen vacancies on CeO_2_ surface serve as active center towards CO_2_ hydrogenation to methanol, where the catalytic activity is highly correlated with the oxygen vacancies concentration^41^. This evokes us to design a suitable metal-support interface structure with synergistic catalysis effect, so as to simultaneously promote catalytic activity and stability for DRM reaction and further reveal the structure-property correlation at molecular/atomic scale.

**Fig. 1:**
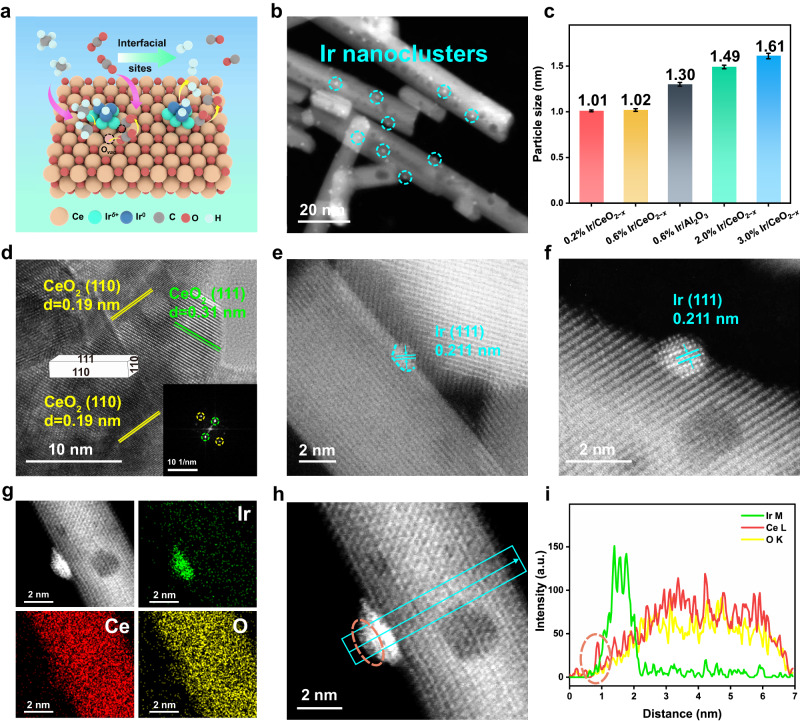
Microstructure and morphology studies of Ir/CeO_2−*x*_ samples. **a** Schematic illustration of Ir/CeO_2−*x*_ samples. **b**, **d** TEM and HR-TEM images of 0.6% Ir/CeO_2−*x*_. **c** Particle size of various Ir/CeO_2−*x*_ and Ir/Al_2_O_3_ samples determined by TEM. **e**, **f** High-resolution AC-HAADF-STEM images of 0.2% and 0.6% Ir/CeO_2−*x*_, respectively. **g**, **h** AC-HAADF-STEM image and corresponding EDS mapping of 0.6% Ir/CeO_2−*x*_. **i** Corresponding elemental line scanning of 0.6% Ir/CeO_2−*x*_.

Herein, we report an Ir nanoclusters supported on CeO_2_ catalyst prepared through a facile impregnation-reduction method. HAADF-STEM, *quasi* in situ XPS and in situ XAFS confirm the formation of interface structure (Ir^*δ*+^−O_*v*_−Ce^3+^), whose concentration can be modulated via adjusting the Ir loading. The optimal catalyst 0.6% Ir/CeO_2−*x*_ (Fig. 1a) exhibits high conversions of CH_4_ (~72%) and CO_2_ (~82%) at 700 °C, with a CH_4_ reaction rate of ~973 μmol_CH4_ g_cat_^−1^ s^−1^; and a 100 h stream-on-line test demonstrates a satisfactory stability without obvious deactivation. This is, to the best of our knowledge, preponderant to the state-of-the-art catalysts under similar reaction conditions. Kinetics studies verify that the dissociation of CH_4_ is the rate-determining step in DRM reaction, whose activation energy decreases significantly by ~50 kJ mol^−1^ owing to the interfacial synergistic catalysis. *Operando* investigations (DRIFTS and XAFS), catalytic evaluations and DFT calculations substantiate that the interfacial sites (Ir^*δ*+^−O_*v*_−Ce^3+^) serve as the intrinsic active center: CH_4_ molecule undergoes activation adsorption and dissociation to CH_2_* species and H_2_ at the interfacial Ir^*δ*+^ site, and then CH_2_* experiences oxidation by neighboring oxygen species to generate CH_2_O*, followed by CH_2_O* dehydrogenation to produce CO and H_2_; the concomitant O_*v*_ is replenished by the activation adsorption of C=O group in CO_2_. This interfacial synergistic catalysis not only enhances the catalytic activity for DRM reaction, but also inhibits catalyst deactivation from excessive decomposition of CH_2_* species to carbon deposition.

## Results and discussion

### Structural characterizations of catalysts

Both Ir/Al_2_O_3_ and Ir/CeO_2−*x*_ samples with various Ir loading (0.2%–3%) were prepared via a facile impregnation-reduction method, whose XRD patterns (Supplementary Fig. 1) displayed a series of characteristic reflections indexed to a typical Al_2_O_3_ (JCPDS 77-0396) and CeO_2_ (JCPDS 78-0694) phase, respectively. No recognizable diffractions peaks of Ir or IrO_2_ are found for these samples, implying a highly dispersed Ir species and/or a low Ir content. TEM images of Ir/CeO_2−*x*_ samples (Fig. 1b) show numerous Ir nanoclusters are well dispersed and anchored onto the CeO_2_ nanorods support, in which the mean particle size of Ir increases from ~1.0 nm (0.2% and 0.6% Ir/CeO_2−*x*_) to ~1.5 nm (2% Ir/CeO_2−*x*_) and then to ~1.6 nm (3.0% Ir/CeO_2−*x*_) (Fig. 1c and Supplementary Fig. 4−7). Accordingly, the dispersion of Ir (*D*_Ir_, Supplementary Table 1) decreases gradually from 82% (0.2% Ir/CeO_2−*x*_) to 39% (3.0% Ir/CeO_2−*x*_). From the local magnification HR-TEM images (Fig. 1d and Supplementary Fig. 3−7), two clear crystalline phases are identified as ~0.19 and ~0.31 nm, respectively, corresponding to the (110) and (111) planes of CeO_2_ nanorods support. As shown in Supplementary Fig. 8, the (110) facet is predominantly exposed accompanied with minor (111) facet, consistent with the previous reports^41,42^. As a control sample, 0.6% Ir/Al_2_O_3_ displays a larger particle size (~1.3 nm) and a lower dispersion (~65%) relative to 0.6% Ir/CeO_2−*x*_, along with lattice spacings of ~0.19 and ~0.23 nm ascribed to (400) and (311) planes of Al_2_O_3_^43^. In addition, the aberration-correction high-angle annular dark-field scanning transmission electron microscopy (AC-HAADF-STEM) was conducted to explore detailed structure of Ir/CeO_2−*x*_. As shown in Fig. 1e and f, a clear lattice fringe (~0.211 nm) indexed to Ir(111) plane is observed on the surface of CeO_2_ for both the 0.2% and 0.6% Ir/CeO_2−*x*_ samples. Moreover, the energy dispersive spectroscopy (EDS) elemental mapping and elemental line scanning of 0.6% Ir/CeO_2−*x*_ sample (Fig. 1g−i) show a partial coating of CeO_2_ on the surface of Ir cluster, indicating the formation of interfacial structure between Ir and CeO_2_.

**Fig. 2:**
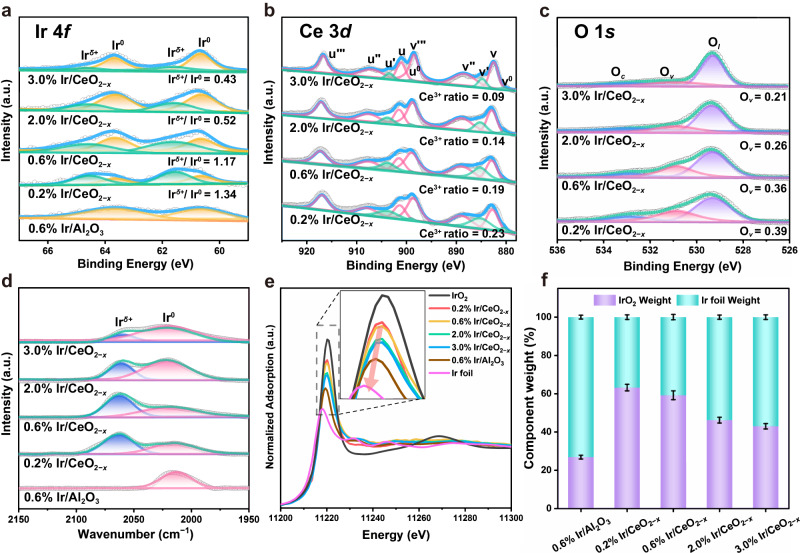
Fine-structure characterizations of Ir/Al_2_O_3_ and Ir/CeO_2−*x*_ samples. **a**–**c** *Quasi* in situ XPS of Ir 4*f*, Ce 3*d* an O 1*s* for Ir/Al_2_O_3_ and Ir/CeO_2−*x*_ samples with various Ir loading. **d** In situ CO-DRIFTS spectra on the surface over Ir/Al_2_O_3_ and various Ir/CeO_2−*x*_ samples. **e** Ir L_3_-edge XANES spectra and (**f**) diagram of the linear combination fitting (LCF) results for various samples.

*Quasi* in situ XPS spectra were performed to investigate the electronic structure of surface Ir species. As shown in Fig. 2a, the Ir/Al_2_O_3_ sample displays two peaks at 60.6 eV (Ir 4*f*_7/2_) and 63.6 eV (Ir 4*f*_5/2_) corresponding to the Ir^0^ species. In contrast, for the four Ir/CeO_2−*x*_ samples, besides the same Ir^0^ peaks, two additional strong peaks at 61.6 eV (Ir 4*f*_7/2_) and 64.6 eV (Ir 4*f*_5/2_) are found, which are attributed to the Ir^*δ*+^ species^44–46^. This indicates the electron transfer from Ir species to CeO_2_ support at the interface via the electronic metal-support interaction (EMSI), which is absent in the Ir/Al_2_O_3_ sample. With the increase of Ir content, the ratio of Ir^*δ*+^/(Ir^*δ*+^+Ir^0^) declines gradually from 57% (0.2% Ir/CeO_2−*x*_) to 30% (3.0% Ir/CeO_2−*x*_) (Supplementary Table 2), as a result of the decreased Ir dispersion degree. Furthermore, in situ CO-DRIFTS is implemented to investigate the configuration of Ir species (Fig. 2d), from which a broad band centered at ~2020 cm^−1^ due to the linear CO at Ir^0^ site is found for the Ir/Al_2_O_3_ sample. Notably, in the case of Ir/CeO_2−*x*_ samples, both the linear adsorption of CO at Ir^0^ (~2020 cm^−1^) and gem-dicarbonyl species adsorption at Ir^*δ*+^ (~2060 cm^−1^) are observed^44,45,47^. With the increment of Ir loading, according to the Gaussian peak fitting results, the relative peak intensity of Ir^*δ*+^/(Ir^*δ*+^+Ir^0^) displays an obvious decrease from 0.2% Ir/CeO_2−*x*_ (56%) to 3.0% Ir/CeO_2−*x*_ (23%) (Supplementary Table 3), in accordance with the variation tendency of Ir^*δ*+^/(Ir^*δ*+^+Ir^0^) in the XPS results.

X-ray absorption near-edge structure (XANES) measurements at normalized Ir L_3_-edge were implemented to analyze the electronic state and coordination fine structure. As shown in Fig. 2e, the white line peaks of Ir/CeO_2−*x*_ and Ir/Al_2_O_3_ samples are located between Ir foil and IrO_2_ reference, suggesting the existence of positively charged Ir species. Moreover, the intensity of white line declines gradually from 0.2% Ir/CeO_2−*x*_ to 3.0% Ir/CeO_2−*x*_ and then to Ir/Al_2_O_3_, indicating the decrease in the oxidation state of Ir^*δ*+^ species (reduced interfacial electron transfer) along with the increase in metallic Ir^0^. The Fourier transforms of the extended X-ray absorption fine spectra (EXAFS) in the R space (Supplementary Fig. 10) show that all these samples exhibit coexistent of Ir−O scattering (~1.5 Å) and Ir−Ir scattering (~2.5 Å). Accordingly, we conducted the linear combination fitting (LCF) of XANES (Fig. 2f) to determine the Ir species composition in these samples. The control sample Ir/Al_2_O_3_ displays a low Ir^4+^ atomic ratio of 27%. In contrast, the Ir^4+^ is predominant for the Ir/CeO_2−*x*_ samples, in which the Ir^4+^ atomic ratio of 0.2% and 0.6% Ir/CeO_2−*x*_ samples are 63% and 59% (Supplementary Table 4), respectively; whilst the Ir^0^ plays a leading role for the 2.0% and 3.0% Ir/CeO_2−*x*_ samples, as a result of the increased particle size of Ir. The average oxidation state of iridium species is calculated based on the results from LCF analysis, which gives the following sequence: 0.2% Ir/CeO_2−*x*_ (+2.5) > 0.6% Ir/CeO_2−*x*_ (+2.4) > 2.0% Ir/CeO_2−*x*_ (+1.8) > 3.0% Ir/CeO_2−*x*_ (+1.7) > 0.6% Ir/Al_2_O_3_ (+1.1).

Then, we used *quasi* in situ XPS to study the defective sites of Ir/CeO_2−*x*_ samples, which contribute to mediate the CO_2_ activation. For the pristine CeO_2_ support (Supplementary Fig. 13), the spectra of Ce 3*d* show six strong peaks at 882.9, 889.0, 898.6 eV (marked as v, v″, and v‴, respectively) and 901.5, 907.6, 917.2 eV (marked as u, u″, and u‴, respectively) assigned to 3*d*^10^4*f* ^0^ state of Ce^4+^ species, with a spin-orbit splitting of about 18.6 eV. In terms of Ir/CeO_2−*x*_ samples (Fig. 2b), four peaks appear at 880.5 eV, 885.2 eV, 899.1 eV, and 903.8 eV (marked as v^0^, v′, u^0^, and u′, respectively) belonging to 3*d*^10^4*f* ^1^ state of Ce^3+^ ^41,48^. The relative concentration of surface Ce^3+^, calculated by Ce^3+^/(Ce^3+^ + Ce^4+^) based on corresponding peak areas (Supplementary Table 2), decreases gradually from 23% (0.2% Ir/CeO_2−*x*_) to 10% (3.0% Ir/CeO_2−*x*_). Moreover, the oxygen vacancies of Ir/CeO_2−*x*_ and Ir/Al_2_O_3_ samples were further analyzed via deconvolution of *quasi* in situ O 1*s* XPS spectra. As shown in Fig. 2c, three peaks are found at 529.3, 530.8 and above 532.8 eV, which are assigned to the lattice oxygen (O_*l*_), oxygen vacancies (O_*v*_) and other weakly bound oxygen species (O_*c*_, such as hydroxyl oxygen or chemisorbed oxygen species), respectively^48,49^. The relative ratio of oxygen vacancy (O_*v*_) calculated as O_*v*_/(O_*l*_ + O_*v*_ + O_*c*_) ranks in the following order: 0.2% Ir/CeO_2−*x*_ (39%) > 0.6% Ir/CeO_2−*x*_ (36%) > 2.0% Ir/CeO_2−*x*_ (26%) > 3.0% Ir/CeO_2−*x*_ (21%) (Supplementary Table 2), in agreement with the tendency of Ce^3+^/(Ce^3+^ + Ce^4+^) ratio. Therefore, in situ CO-DRIFTS, XAFS and *quasi* in situ XPS results notarize the formation of interface structure (Ir^*δ*+^−O_*v*_−Ce^3+^) originating from SMSI, whose relative concentration decreases with increment of Ir loading.

### Catalytic evaluations

**Fig. 3:**
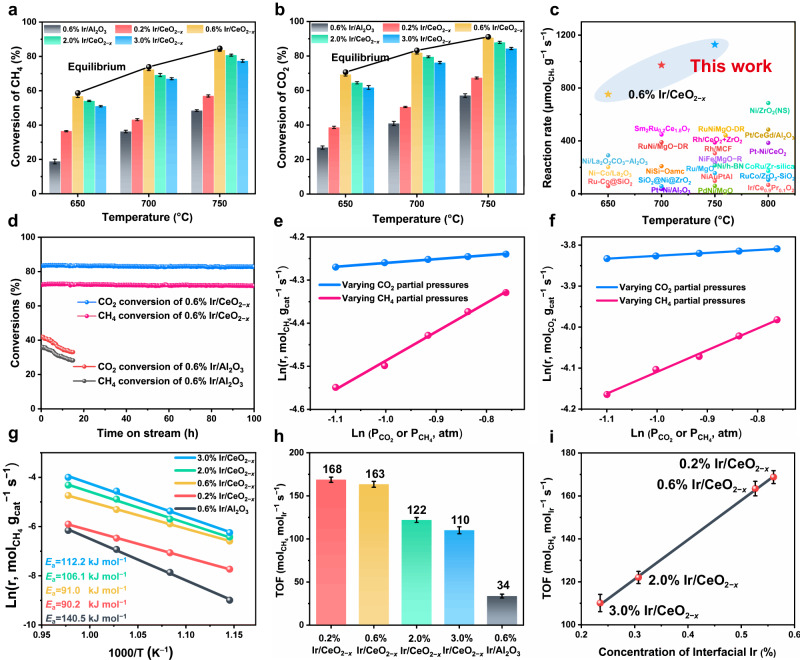
Catalytic performance of various catalysts. **a**, **b** CH_4_ conversion and CO_2_ conversion over various samples at 650, 700, and 750 °C. **c** Comparison study on mass specific activity between 0.6% Ir/CeO_2−*x*_ and other typical catalysts used in DRM reaction^64–79^. **d** Stability test of 0.6% Ir/CeO_2−*x*_ and 0.6% Ir/Al_2_O_3_ at 700 °C for DRM. Evaluated conditions: CH_4_/CO_2_/N_2_ = 20/20/5 mL min^−1^, GHSV = 240000 mL g^−1^ h^−1^. **e**, **f** Correlation of CH_4_ or CO_2_ partial pressure on the reaction rates of CH_4_ and CO_2_. **g** Kinetic studies and calculated activation energy (*E*_a_) of CH_4_ over various catalysts. **h** Intrinsic TOF over various catalysts within the catalytic dynamic range. **i** TOF as a function of interfacial Ir concentration calculated by CO-DRIFTS results.

The preceding samples were evaluated for DRM under a gas hourly space velocity as high as 240000 mL g^−1^ h^−1^ at atmospheric pressure. As shown in Fig. 3a and b, both the CH_4_ and CO_2_ conversions as a function of reaction temperature show a positive correlation for these samples, duo to the strong endothermic characteristic. The control sample 0.6% Ir/Al_2_O_3_ gives a normal catalytic performance towards DRM reaction; whilst the catalytic performance of Ir/CeO_2−*x*_ samples improve significantly. Notably, the catalytic activity exhibits a volcanic curve at each reaction temperature along with the increase of Ir loading: an increase from 0.2% Ir/CeO_2−*x*_ to 0.6% Ir/CeO_2−*x*_ (the maximum value) is present, followed by a slight descend to 2.0% Ir/CeO_2−*x*_ and 3.0% Ir/CeO_2−*x*_. As for the optimal 0.6% Ir/CeO_2−*x*_ sample, both the CH_4_ and CO_2_ conversions reach up to the thermodynamic equilibrium, and the reaction rate is 3−20 times higher than previously reported studies under similar reaction conditions within 650−750 °C (Fig. 3c and Supplementary Table 5)^19,50^. Specifically, the 0.6% Ir/CeO_2−*x*_ catalyst exhibits high conversions of CH_4_ (72%) and CO_2_ (82%) with a CH_4_ reaction rate of ~973 μmol_CH4_ g_cat_^−1^ s^−1^ at a relatively moderate temperature (700 °C), which are precedent to the state-of-the-art catalysts^6,7,9,16,20,24,25,49–51^. In addition, the long-term stability test displays a rapid deactivation for the Ir/Al_2_O_3_ sample within 15 h due to Ir agglomeration and carbon deposition at 700 °C (Supplementary Fig. 16 and 17). In contrast, both the CH_4_ and CO_2_ conversions of 0.6% Ir/CeO_2−*x*_ catalyst remain almost unchanged within 100 h on stream (Fig. 3d). Moreover, the used 0.6% Ir/CeO_2−*x*_ catalyst does not show obvious structural change compared with the fresh sample, verified by TEM, XPS and in situ CO-DRIFTS (Supplementary Fig. 18−20), indicating a satisfactory stability in DRM reaction. The results above demonstrate excellent performance of 0.6% Ir/CeO_2−*x*_ catalyst, which shows potential application in industrial applications.

Furthermore, we performed kinetic studies on CH_4_ and CO_2_ activation as well as the rate-determining step in DRM system. Firstly, the effects of external and internal diffusion limitation have been eliminated under the aforementioned reaction conditions^34,50,52^. On this basis, the kinetic experimental data were studied via setting a stationary partial pressure of one reactant whilst changing the other partial pressure (Fig. 3e, f), and the obtained results were calculated for kinetic parameters and were shown in Supplementary Table 6. The reaction rate over 0.6% Ir/CeO_2−*x*_ catalyst displays a linear positive correlation with the partial pressure of CH_4_ and CO_2_. Nevertheless, the calculated reaction order with respect to CH_4_ (~0.67 and ~0.53) is significantly higher than that of CO_2_ (~0.09 and ~0.07), indicating that the CH_4_ activation is critical to the reaction kinetics, consistent with previous studies^50,52,53^. Moreover, the apparent activation energy (*E*_a_) of CH_4_ over 0.6% Ir/CeO_2−*x*_ is 91 kJ mol^−1^, much larger than that of CO_2_ (70 kJ mol^−1^) (Fig. 3g and Supplementary Fig. 22 and 23). The results verify that the CH_4_ dissociation entails a higher energy barrier and serves as the rate-determining step in this catalytic system. Notably, the *E*_a_ value on 0.6% Ir/CeO_2−*x*_ catalyst shows a marked decline by 35% relative to the 0.6% Ir/Al_2_O_3_ sample, which indicates the interfacial sites play a critical role in activating reactant molecule. In addition, the intrinsic TOF of CH_4_ is evaluated at a low conversion (below 15%, Fig. 3h), which gives a decrease order as follows: 0.2% Ir/CeO_2−*x*_ (168 mol_CH4_ mol_Ir_^−1^ s^−1^) > 0.6% Ir/CeO_2−*x*_ (163 mol_CH4_ mol_Ir_^−1^ s^−1^) > 2.0% Ir/CeO_2−*x*_ (122 mol_CH4_ mol_Ir_^−1^ s^−1^) > 3.0% Ir/CeO_2−*x*_ (110 mol_CH4_ mol_Ir_^−1^ s^−1^). To further the reveal correlation of intrinsic active site and structure-property, the intrinsic TOF of CH_4_ is plotted as a function of interfacial Ir^*δ*+^ concentration (based on the results of in situ CO-DRIFTS), from which an approximative linear relationship is present (Fig. 3i). Furthermore, a positive correlation between intrinsic TOF and surface Ce^3+^ ratio (Supplementary Fig. 24) or surface oxygen vacancy ratio (Supplementary Fig. 25) is also demonstrated. The results above elucidate that the Ir^*δ*+^−O_*v*_−Ce^3+^ interfacial sites serve as the intrinsic active center towards DRM reaction, accounting for the prominent catalytic performance.

#### Catalytic mechanism

**Fig. 4:**
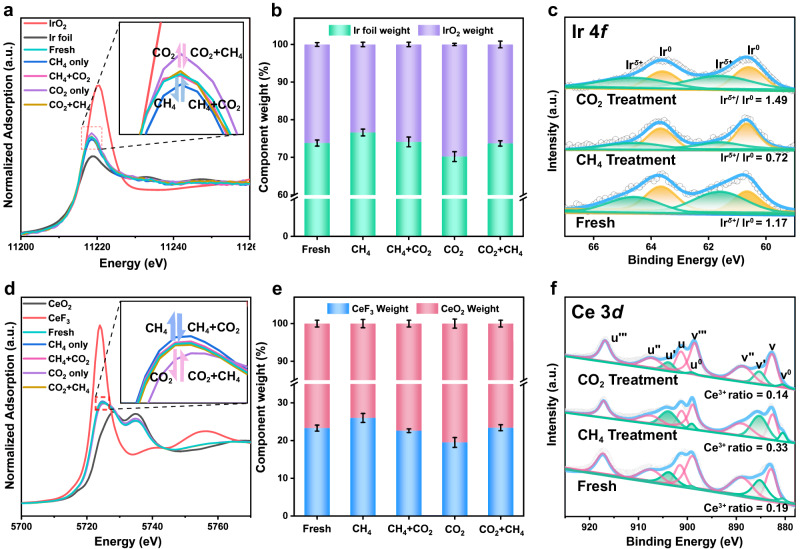
Local coordination structure and surface structure of 0.6% Ir/CeO_2_ during DRM reaction. **a**, **b** In situ*/operando* normalized XANES at Ir L_3_-edge and diagram of the linear combination fitting (LCF) results of 0.6% Ir/CeO_2−*x*_ with CH_4_, CO_2_ and CH_4_ + CO_2_ treatment, respectively. **d**, **e** Ce L_3_-edge and diagram of the linear combination fitting (LCF) results of 0.6% Ir/CeO_2−*x*_ with CH_4_, CO_2_ and CH_4_ + CO_2_ treatment, respectively. **c**, **f** *Quasi* in situ XPS spectra of Ir 4*f* and Ce 3*d* for the fresh 0.6% Ir/CeO_2−*x*_ and the same catalyst after CH_4_ or CO_2_ treatment at 700 °C.

In situ*/operando* XANES of Ir L_3_-edge and Ce L_3_-edge combined with *quasi* in situ XPS were applied to reveal the dynamic variation of fine structure and electronic interaction at interfacial sites under the catalytic reaction. During the measurement, CH_4_ and CO_2_ was introduced into the reaction cell in turn, and the catalytic reaction was triggered at 700 °C via injecting the second reactant gas, so as to observe the formation and variation of interface structure (Ir^*δ*+^−O_*v*_−Ce^3+^). When CH_4_ is introduced alone, the white line peaks of Ir and Ce shift close to the reference Ir foil and CeF_3_ (Fig. 4a, d), respectively, indicating a decline in valence states of Ir and Ce (Fig. 4b, e). The corresponding variations in XPS spectra of Ir 4*f* and Ce 3*d* are also observed (Fig. 4c, f): the Ir^*δ*+^/(Ir^*δ*+^+Ir^0^) ratio decreases whilst the Ce^3+^/(Ce^3+^ + Ce^4+^) ratio and O_*v*_ increase. This implies the occurrence of CH_4_ dissociation to CH_x_^*^ species, which then combines with surface reactive O to generate more Ir^*δ*+^−O_*v*_−Ce^3+^ interface sites. After the injection of CO_2_, the white line peaks of Ir and Ce shift back to their original position, indicating the replenishment of O_*v*_ by CO_2_. In Fig. 4a, d, as CO_2_ is introduced alone, the white line peaks of Ir and Ce move close to the reference IrO_2_ and CeO_2_, respectively (elimination of primary O_*v*_); and XPS results show the increased valence states of Ir and Ce accompanied with reduced Ce^3+^/(Ce^3+^ + Ce^4+^) ratio and O_*v*_ (Fig. 4b, e). Afterwards, the subsequent CH_4_ flowing induces the recovery of Ir and Ce white line peaks to their original position, corresponding to the CH_4_ dissociation assisted with surface reactive oxygen species.

**Fig. 5:**
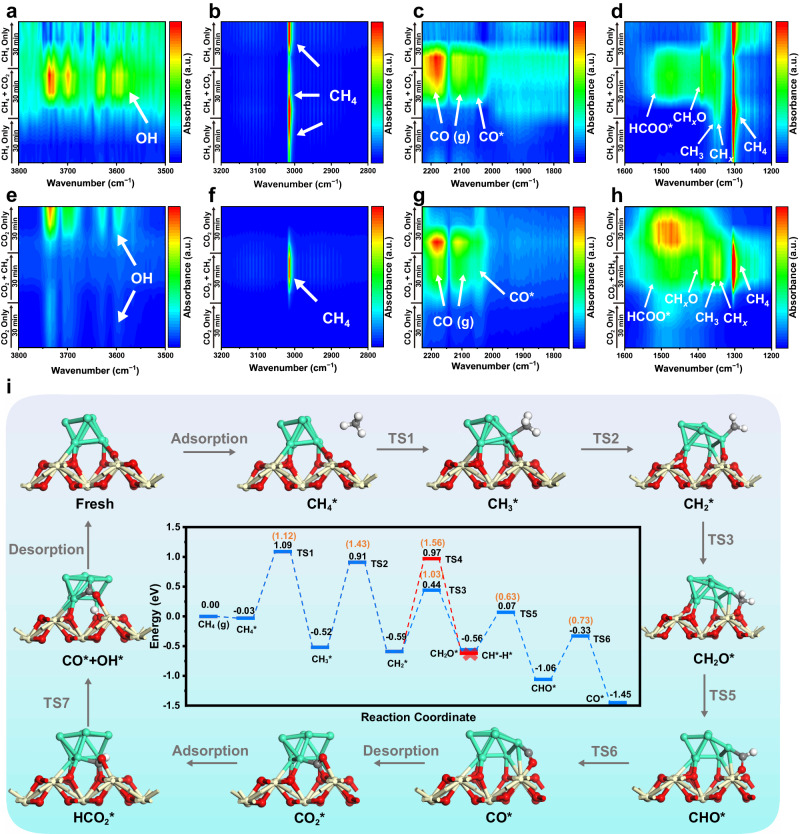
In situ*/operando* DRIFTS spectra and DFT calculations of DRM reaction on 0.6% Ir/CeO_2−*x*_. In situ*/operando* DRIFTS spectra over 0.6% Ir/CeO_2−*x*_ at 700 °C after in-situ pretreatment and He purging, followed by exposure to: (**a**−**d**) first CH_4_ atmosphere, subsequent CH_4_ + CO_2_ and then CH_4_ atmosphere for 30 min, respectively; (**e**−**h**) first CO_2_ atmosphere, subsequent CO_2_ + CH_4_ and then CO_2_ atmosphere for 30 min, respectively. **i** Schematic illustration for DRM reaction at the interface of Ir/CeO_2−*x*_. Ir, green; Ce, yellow; C, gray; O, crimson; H, white. The inset shows potential energy profile of CH_4_ decomposition by Ir/CeO_2−*x*_(110). ‘TS’ represents a transition state. The black and orange numbers represent the adsorption energies and energy barriers of the elementary steps, respectively.

In situ*/operando* DRIFTS experiments of reactants were carried out to further identify the intermediate species and monitor the evolution of dynamic reaction process at the interface structure Ir^*δ*+^−O_*v*_−Ce^3+^ (Fig. 5a−h). When CH_4_ is introduced individually into the reactor at 700 °C, in addition to the gas phase CH_4_ at ~3016 and ~1304 cm^−1^, another two bands at ~1330 and ~1350 cm^−1^ corresponding to the deformation vibration of CH_x_* and CH_3_* are observed^4,20,51^, respectively, due to the activation adsorption and dissociation of CH_4_ at interface Ir^*δ*+^ sites. Subsequently, with the injection of CO_2_, two strong peaks located at ~2360 and ~1550 cm^−1^, as well as another broad one at ~3750−3550 cm^−1^ appear, which are attributed to the gas phase CO_2_, the monodentate carbonate species (HCOO*) and surface hydroxyl group (OH*), respectively (Fig. 5b−d and Supplementary Figs. 28, 29)^16,20,54,55^. Notably, another IR band assigned to the CH_*x*_O* species is found at ~1390 cm^−1^, accompanied with the weakened bands of CH_4_ and CH_x_* species^56,57^. This is probably due to the oxidation of CH_x_* by reactive oxygen species originating from CO_2_ dissociation. In addition, three bands between ~2200 and ~2000 cm^−1^ are detected, which are ascribed to gaseous CO and adsorbed CO* at Ir^*δ*+^, respectively (Fig. 5c)^44–46^. Once the atmosphere is switched from the mixture gas (CO_2_ and CH_4_) to individual CH_4_, the bands of gas phase CO_2_ weaken firstly, and then the bands assigned to CH_*x*_O*, HCOO*, OH* and CO species disappear gradually accompanied with the enhancement of CH_4_ and CH_x_* peaks. This demonstrates the oxygen-containing species (CH_*x*_O*, HCOO*, OH*) serves as important intermediate, whose consumption can be reproduced by CO_2_ at interface O_*v*_.

Next, we changed the study paradigm, in which CO_2_ is injected into the reactor firstly under the same conditions. Accordingly, the bands assigned to CO_2_ is observed (Supplementary Fig. 30 and Fig. 5e). With the subsequent flowing of CH_4_, the bands of CH_4_, CH_x_* and CH_3_* species are found (Fig. 5g, h), followed by the emergence of CH_*x*_O* and CO peaks as well as the weakened OH* band. This verifies the significance of CH_*x*_O* species originating from the reaction between CH_x_* and surface oxygen species, in accordance with the results of Fig. 5a−d. O*perando* investigations above (XAFS and DRIFTS) substantiate that the interface structure (Ir^*δ*+^−O_*v*_−Ce^3+^) serves as the intrinsic active center with a crucial synergistic effect: Ir^*δ*+^ promotes the activation adsorption of CH_4_ molecule whilst CO_2_ dissociation occurs at the Ce^3+^−O_*v*_ site, followed by the formation of the key intermediate (CH_*x*_O* species).

To in-depth explore the decisive role of Ir^*δ*+^−O_*v*_−Ce^3+^ interfacial sites in the reaction process, DFT calculations were investigated on Ir_7_/CeO_2−*x*_ model based on the experimental results (Supplementary Fig. 31). As shown in Fig. 5i and Supplementary Fig. 32, firstly, CH_4_ molecule undergoes adsorption at the interfacial Ir^*δ*+^ of Ir_7_/CeO_2−*x*_ (110) with a small adsorption energy (−0.03 eV); then, the C–H bond cleavage of CH_4_ occurs to generate CH_3_* (TS1: 1.12 eV). Afterwards, the CH_3_* species experiences dehydrogenation process which shows an energy barrier of 1.43 eV, excluding the oxidation of CH_3_* to CH_3_O* with a large steric hindrance. Subsequently, two possible steps are involved: (1) CH_2_* oxidation to CH_2_O* and (2) CH_2_* dehydrogenation to CH*. However, the former displays a much lower energy barrier (TS3: 1.03 eV) than the latter (TS4: 1.56 eV), in agreement with the formation of CH_*x*_O* intermediate verified by the *operando* DRIFTS results. This step is crucial, which inhibits excessive decomposition of CH_2_* species to carbon deposition. The next dehydrogenation of CH_2_O* to CHO* (TS5) and CO (TS6) shows normal activation barriers of 0.63 and 0.73 eV, respectively. Finally, the produced CO undergoes desorption from the O_*v*_ and the remaining four active hydrogen form into two H_2_ molecules (Supplementary Fig. 32). Meanwhile, CO_2_ molecule experiences dissociation adsorption at the O_*v*_ on the surface with an adsorption energy of −1.85 eV and an energy barrier of 0.7 eV (Supplementary Fig. 33 and 34), with the formation of active oxygen species that participates in the CH_2_* oxidation to CH_2_O*. According to the calculation results, the dehydrogenation of CH_3_* species to CH_2_* gives the highest energy barrier (1.43 eV), which is determined as the rate-determining step of DRM reaction, in accordance with the experimental results (Fig. 3e−f). In addition, a comparative study between Ir_7_/CeO_2−*x*_ and Ir_7_/Al_2_O_3_ shows that the reaction energy barrier of rate-determiningstep in the former case (1.43 eV) is significantly lower than the latter one (Supplementary Figs. 35, 36: 2.88 eV), demonstrating the essential contributions of interface sites (Ir^*δ*+^−O_*v*_−Ce^3+^), in well agreement with the catalytic performance in Fig. 3a−h.

In summary, we report an Ir/CeO_2−*x*_ catalytic system with metal–support interface structure towards DRM reaction. The obtained 0.6% Ir/CeO_2−*x*_ catalyst exhibits exceptional conversions of CH_4_ (72%) and CO_2_ (82%), a CH_4_ reaction rate of ~973 μmol_CH4_ g_cat_^−1^ s^−1^ and a satisfactory service stability within 100 h at a relatively low temperature (700 °C). A joint investigation based on HAADF-STEM, *quasi* in situ XPS and in situ XAFS confirms the formation of interface structure (Ir^*δ*+^−O_*v*_−Ce^3+^), whose concentration can be modulated via adjusting the Ir loading. *Operando* investigations (DRIFTS and XAFS), catalytic evaluations and DFT calculations substantiate that the interfacial sites (Ir^*δ*+^−O_*v*_−Ce^3+^) serve as the intrinsic active center to facilitate the dissociation of CH_4_ (the rate-determining step) and the oxidation of CH_2_* to CH_2_O*; the concomitant O_*v*_ can be replenished by the activation adsorption of CO_2_. This interfacial synergistic catalysis plays a crucial role in boosting the catalytic performance and inhibiting deactivation, which paves a way for the design of other high-performance heterogeneous catalysts towards structure-sensitive reactions.

## Methods

### Chemicals and materials

Analytical grade chemical reagents were purchased in Aladdin company and used directly without further purification, including: Ce(NO_3_)_3_·6H_2_O, NaOH, Al_2_O_3_, and H_2_IrCl_6_·6H_2_O. Deionized water was adopted in all experiment steps.

### Preparation of catalysts

CeO_2_ nanorods were prepared via a hydrothermal method reported by our group^42^. Typically, Ce(NO_3_)_3_ solution (0.4 M, 20 mL) and NaOH solution (6.8 M, 140 mL) were fully mixed with vigorous stirring for 30 min at room temperature. The obtained milky slurry was placed into a 200 mL sealed Teflon autoclave for 24 h at 100 °C. After filtering, washing thoroughly, and drying at 65  °C for 18 h, the sample was calcined in air at 500 °C with a heating rate of 10 °C min^−1^ for 4 h to obtain the CeO_2_ nanorods support. CeO_2_ (0.5 g) was dispersed into deionized water (20 ml) and H_2_IrCl_6_·6H_2_O aqueous solution (0.022 g mL^−1^; 0.105, 0.315, 1.050, 1.575 mL, respectively) was slowly dripped into above solution with vigorous stirring for various Ir loading samples. After 8 h of reaction, the resulting precipitate was centrifuged, washed thoroughly with deionized water and ethanol, followed by drying at 60 °C for 12 h. Before the DRM reaction, the sample was pre-treated at 750 °C for 3 h in a gaseous mixture of CH_4_ and CO_2_ (1:1, v/v; total flow rate: 50 mL min^−1^). As a reference, the Ir/Al_2_O_3_ sample was prepared via the same method described above by using Al_2_O_3_ as the support, in which the pre-treated steps are in accordance with those of Ir/CeO_2−*x*_ samples.

### Characterizations

X-ray diffraction (XRD) experiments were carried out with Bruker D8 Advance diffractometer. The elemental content was determined by Shimadzu ICPS-7500 equipment. The morphology and structure of catalysts were studied on JEOL JEM-2010 high-resolution transmission electron microscope. AC-HAADF-STEM images and EDS mapping data were performed on JEOL JEM-ARM200F. The CO pulses chemisorption experiments were conducted on Micromeritics Autochem II 2920. *Quasi* in situ XPS measurements were recorded on Kratos Axis Ultra DLD Instrument. The pre-treated sample was placed in a glove box and transferred into a sample rod in a N_2_ atmosphere. In situ*/Operando* XAFS at Ir L_3_-edge and Ce L_3_-edge measurements were recorded at the beamline BL11B of the Shanghai Synchrotron Radiation Facility (SSRF), Shanghai Institute of Applied Physics, Chinese Academy of Sciences (CAS). In situ*/operando* DRIFTS were studied on a Bruker TENSOR II infrared spectrometer with a MCT detector. The detailed experimental methods are present in the Supplementary Information.

### DFT calculations

The density functional theory (DFT) calculations based on first-principle methodology were investigated using the Vienna ab initio simulation package (VASP 5.4.4)^58,59^. Generalized gradient approximation (GGA) of PBE functional was applied to describe the exchange and correlation energy. Grimme’s DFT-D3 method and projector augmented wave (PAW) method were employed to illustrate the effect of van der Waals interaction and to depict the core electrons, respectively^60,61^. The climbing image nudged elastic band (CI-NEB) method was employed to determine reaction transition states^62,63^.

### Supplementary information

Supplementary Information

Peer Review File

### Source data

Source Data

## Data Availability

The primary data that support the plots within this paper and other finding of this study are available from the corresponding author on reasonable request. Source data are provided with this paper.
