# Supplementary material for: Facilitating the dry reforming of methane with interfacial synergistic catalysis in an Ir@CeO2−x catalyst
Source: Nat Commun. 2024 May 4;15:3765. doi: 10.1038/s41467-024-48122-6 (PMC11069590; doi:10.1038/s41467-024-48122-6)
Supplement: Supplementary file 1 — Supplementary Information [file 41467_2024_48122_MOESM1_ESM.pdf]

Supplementary Information

Facilitating the dry reforming of methane with interfacial synergistic catalysis in an Ir@CeO_{2-x} catalyst

Hui Wang,[1] Guoqing Cui,*[2] Hao Lu,[1] Zeyang Li,[1] Lei Wang,[1,3] Hao Meng,[1,3] Jiong Li,[4] Hong Yan,[1] Yusen Yang,*[1,3] and Min Wei*[1,3]

[1] State Key Laboratory of Chemical Resource Engineering, Beijing Advanced Innovation Center for Soft Matter Science and Engineering, Beijing University of Chemical Technology, Beijing 100029, P. R. China

[2] State Key Laboratory of Heavy Oil Processing, China University of Petroleum (Beijing), Beijing 102249, P. R. China

[3] Quzhou Institute for Innovation in Resource Chemical Engineering, Quzhou 324000, P. R. China

[4] Shanghai Synchrotron Radiation Facility, Shanghai Institute of Applied Physics, Chinese Academy of Sciences, Shanghai 201204, P. R. China

Author Information

* Corresponding authors. Tel: +86-10-64412131; Fax: +86-10-64425385.

E-mail addresses: cui@cup.edu.cn (G. Cui); yangyusen@buct.edu.cn (Y. Yang); weimin@mail.buct.edu.cn (M. Wei).

Supplementary methods

Catalytic evaluations. The catalytic performance towards dry reforming of methane (DRM) reaction was studied in stainless steel fix-bed reaction equipment with 8.0 mm interior diameter and 600 mm length at atmospheric pressure. Typically, 10 mg of catalyst sample was installed in the reactor tube and pre-treated at 750 °C for 3 h in a gaseous mixture of CH_4 and CO_2 (1:1, v/v; total flow rate: 50 mL min^{-1}), and then cooled to room temperature in N_2 atmosphere. The catalytic evaluation was then conducted by using a mixture gas of $CH_4/CO_2/N_2$ (20:20:5 mL min^{-1}, GHSV=240000 mL g^{-1} h^{-1}) in the temperature range 650−750 °C, with a 3 h balance time for steady state at each temperature point. The composition of products was analyzed with an online gas chromatograph (GC-2014C, Shimadzu Company) equipped with a TDX-01 column and TCD detector. CH_4 conversion, CO_2 conversion and reaction rate are calculated according to the following formula:

$$Con_{CH_4}(\%) = \frac{Moles\ of\ \mathrm{CH_4}(in) - Moles\ of\ \mathrm{CH_4}(out)}{Moles\ of\ \mathrm{CH_4}(in)} \times 100\% \quad (1)$$

$$Con_{CO_2}(\%) = \frac{Moles\ of\ \mathrm{CO_2}(in) - Moles\ of\ \mathrm{CO_2}(out)}{Moles\ of\ \mathrm{CO_2}(in)} \times 100\% \quad (2)$$

$$\text{Reaction rate}\ \left(\mu\mathrm{mol_{CH_4}}\ \mathrm{g_{cat}^{-1}}\ \mathrm{s^{-1}}\right) = \frac{Molars\ of\ \mathrm{CH_4}\ converted}{Mass\ of\ catalyst\ \times Reaction\ time} \quad (3)$$

The TOF of CH_4 was measured at a low conversion (below 15%) and calculated according to the following formula:

$$\mathrm{TOF}\ \left(\mathrm{mol_{CH_4}}\ \mathrm{mol_{Ir}^{-1}}\ \mathrm{s^{-1}}\right) = \frac{Molars\ of\ \mathrm{CH_4}\ converted}{Molars\ of\ metal\ on\ catalyst\ surface \times Reaction\ time} \quad (4)$$

CO pulses chemisorption measurements. The dispersion degree of metal Ir (*D* (%)) was obtained from CO chemisorption on a Micromeritics AutoChem II 2920 Chemisorption. The sample (100 mg) was pre-treated at 750 °C for 2 h in a mixture gas CH_4/CO_2 atmosphere (1/1, v/v), followed by flushing with high purity He for 1.0 h at 750 °C. Subsequently, the temperature was decreased to 40 °C, and then successive pulses of CO/He (1/99, v/v; 30 mL min^{-1}) were introduced for 30 times until a

saturation adsorption was obtained. The dispersion degree of metal Ir (*D* (%)) was calculated on the basis of CO chemisorption value:

$$D\ (\%) = \frac{SF \times V_{ad} \times M_{Ir}}{m_s \times W_s \times V_m \times d_r} \times 100 \quad (5)$$

Where M_{Ni}, m_s, V_{ad} and W_s are the molecular weight of Ir, the weight of sample (g), the volume of chemisorbed CO and the weight fraction of Ir, respectively. V_m is the molar volume of CO at the standard temperature and pressure (STP); d_r is the reduction degree of Ir; *SF* is the stoichiometric factor, which is assumed as 1:1 for CO: Ir.

***Quasi in situ* XPS measurements.** *Quasi in situ* X-ray photoelectron spectra (XPS) measurements were performed using an apparatus (Kratos Axis Ultra DLD Instrument) with Al Kα radiation (~1486.6 eV). To investigate the elements chemical state, the sample was pre-treated in a mixture gas (CH_4/CO_2 = 1/1; flow rate: 50 mL min^{-1}) at 750 °C (heating rate: 10 °C min^{-1}) for 2 h, followed by N_2 stream for 1 h and cooling to the room temperature. For the XPS testing samples, air is strictly isolated during sample pretreatment and transfer. Typically, the sample was firstly held by quartz wool and was placed in the middle of a quartz tube reactor. After the pre-treated process, the sample was cooled down to room temperature in N_2 flowing. Then, the reactor was sealed and carefully transferred into a N_2 atmosphere glove box, followed by sample preparation and installation in an airtight transport chamber for XPS measurement. The contaminated carbon C 1*s* signal (284.6 eV) was used to calibrate binding energy.

***In situ* DRIFTS measurements**. *In situ* DRIFTS measurements were carried out on a Bruker TENSOR II infrared spectrometer equipped with an MCT narrow-band detector and a specialized *in situ* reaction cell. The sample was pre-treated in a mixture gas (CH_4/CO_2 = 1/1; flow rate: 50 mL min^{-1}) at 750 °C (heating rate: 10 °C min^{-1}) for 1 h, followed by He stream for 1 h and cooling to the room

temperature. As for CO-DRIFTS measurements, the background signal was collected at 25 °C, followed by introducing 5% CO/He (20 mL min^{-1}) into the cell, and DRIFTS spectra were collected until the adsorption spectrum remained unchanged. Finally, the gas flow was switched to a pure He stream (20 mL min^{-1}) to remove the physically adsorbed CO for the collection of CO chemisorption signals. As for reactant-DRIFTS measurements, after the same pre-treated process as above, the temperature was raised to 700 °C in a He stream and then 5% CH_4/He (10 mL min^{-1}) was feed into the reactor for 30 min. Subsequently, another reactant (5% CO_2/He, 10 mL min^{-1}) was introduced into the cell for 30 min, followed by feeding the initial reactant for another 30 min. Next, we changed the study paradigm, in which CO_2 was injected into the reactor firstly under the same conditions followed by the introduction of CH_4.

***In situ* XAFS measurements.** *In situ* X-ray absorption fine structure spectroscopy (XAFS) at the Ir L_3-edge (fluorescence mode) and Ce L_3-edge (transmission mode) was measured at the beamline BL11B of the Shanghai Synchrotron Radiation Facility (SSRF), Shanghai Institute of Applied Physics, Chinese Academy of Sciences (CAS). Typically, the powdered sample was pressed into thin sheet and carefully placed into a reaction microdevice equipped with polyimide windows. Afterwards, the sample was pre-treated in a mixture gas (CH_4/CO_2 = 1/1; flow rate: 50 mL min^{-1}) at 750 °C (heating rate: 10 °C min^{-1}) for 1 h, followed by purging He stream for 1 h and cooling to the room temperature. Subsequently, the temperature was raised to 700 °C in a He stream and 5% CH_4/He (20 mL min^{-1}) was feed into the reactor to trigger the surface reaction. Finally, another reactant (5% CO_2/He, 20 mL min^{-1}) was introduced into the cell. The IFFEFIT 1.2.11 date analysis package (Athena, Artemis, Atoms, and FEFF6) was used for analysis and fitting.

Mears criterion for external diffusion. If $\frac{-r_A'\rho_b Rn}{k_c C_{Ab}} < 0.15$, then the external mass transfer effects

can be neglected [1–3].

$-r_A'$ = reaction rate, kmol kg_{cat}^{-1} s^{-1}

n = reaction order

R = catalyst particle radius, m

ρ_b = bulk density of catalyst bed, kg m^{-3}

ρ_c = solid catalyst density, kg m^{-3}

C_{Ab} = bulk gas concentration of A, kmol m^{-3}

k_c = mass transfer coefficient, m s^{-1}

$\frac{-r_A'\rho_b R n}{k_c C_{Ab}}$ = [1.09 × 10^{-3} kmol kg_{cat}^{-1} s^{-1}] × [493 kg_{cat} m^{-3}] × [1.3 × 10^{-4} m] × [0.67] / [0.128 m s^{-1}]

× [0.045 kmol m^{-3}] = 0.012 < 0.15

Mears for internal diffusion. If $C_{WP} = \frac{-r'_{A(obs)}\rho_c R^2}{D_e C_{As}} < 1$, then the internal mass transfer effects can be neglected.

$-r'_{A(obs)}$ = observed reaction rate, kmol kg_{cat}^{-1} s^{-1}

R = catalyst particle radius, m

ρ_c = solid catalyst density, kg m^{-3}

D_e = effective gas-phase diffusivity, m^2 s^{-1} = $\frac{D_{AB}\phi_p\sigma_c}{\tau}$

where D_{AB} = gas-phase diffusivity, m^2 s^{-1}; ϕ_p = pellet porosity; σ_c = constriction factor

τ = tortuosity

C_{As} = gas concentration of A at the catalyst surface, kmol m^{-3}

$C_{WP} = \frac{-r'_{A(obs)}\rho_c R^2}{D_e C_{As}}$ = [1.09 × 10^{-3} kmol kg_{cat}^{-1} s^{-1}] × [616 kg_{cat} m^{-3}] × [1.3 × 10^{-4} m]^2 / [3.2 × 10^{-6}

$m^2\ s^{-1}] \times [0.045\ kmol\ m^{-3}] = 0.0788 < 1$

Therefore, in this work, both external and internal diffusion for DRM reaction can be neglected.

Computational details. $Ir_7/CeO_{2-x}(110)$ and $Ir_7/Al_2O_3(400)$ models were constructed based on experimental data. The experimental characterization results show that the (110) and (400) crystal planes are the optimal exposed crystallographic surfaces for CeO_2 and Al_2O_3, respectively. The $CeO_2(110)$ surface was represented by a three-layer slab with a $p(2\times3)$ supercell. The $Ir_7/CeO_{2-x}(110)$ model was constructed by loading a Ir_7 cluster on the $CeO_2(110)$ surface with an oxygen vacancy around the clusters. A two-layer slab with a $p(2\times2)$ supercell was used to represent the $Al_2O_3(400)$ surface and the $Ir_7/Al_2O_3(400)$ model was built by loading a Ir_7 cluster on the surface. Adjacent slabs are separated by a vacuum zone with a thickness of 15 Å to avoid periodic interactions perpendicular to the surface. The reaction energies and barriers are calculated as $E_r = E_{FS} - E_{IS}$ and $E_a = E_{TS} - E_{IS}$, where E_{IS}, E_{FS} and E_{TS} are the energies of the corresponding initial state (IS), final state (FS) and transition state (TS), respectively. The convergence criterion for the total energy self-consistent iterations was 10^{-5} eV, and the geometry optimization stopped when the total force on the system was less than 0.05 eV Å^{-1}.

Supplementary data

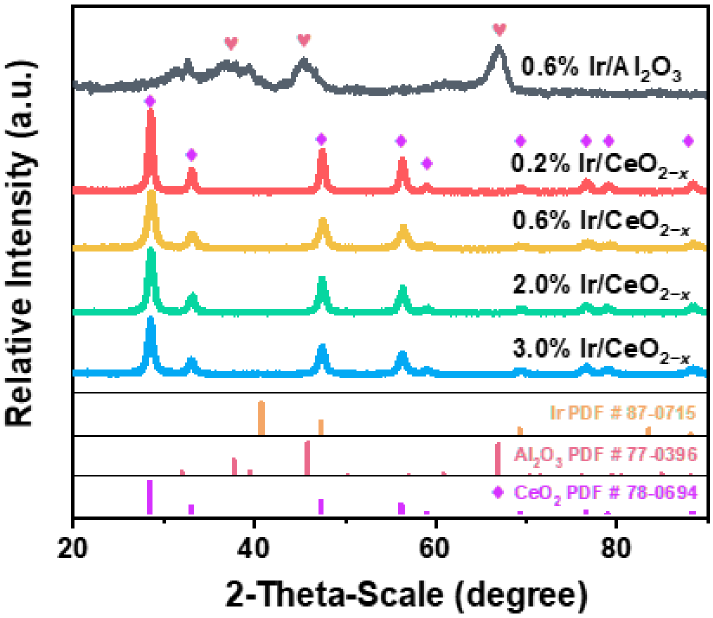

Supplementary Figure 1. XRD patterns of Ir/Al_2O_3 and various Ir/CeO_{2-x} samples.

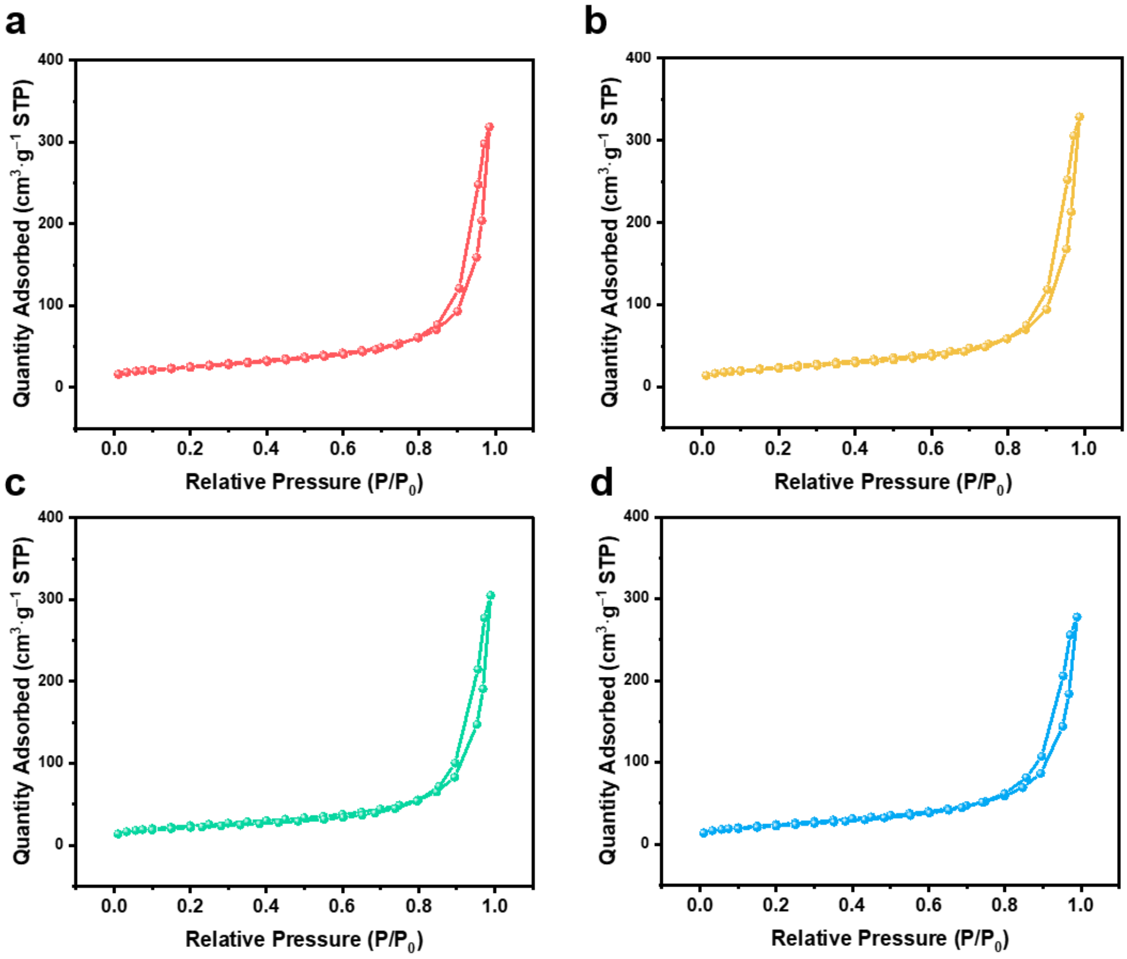

Supplementary Figure 2. a−d Nitrogen adsorption-desorption isotherm of 0.2% Ir/CeO_{2-x}, 0.6% Ir/CeO_{2-x}, 2.0% Ir/CeO_{2-x}, and 3.0% Ir/CeO_{2-x} samples, respectively.

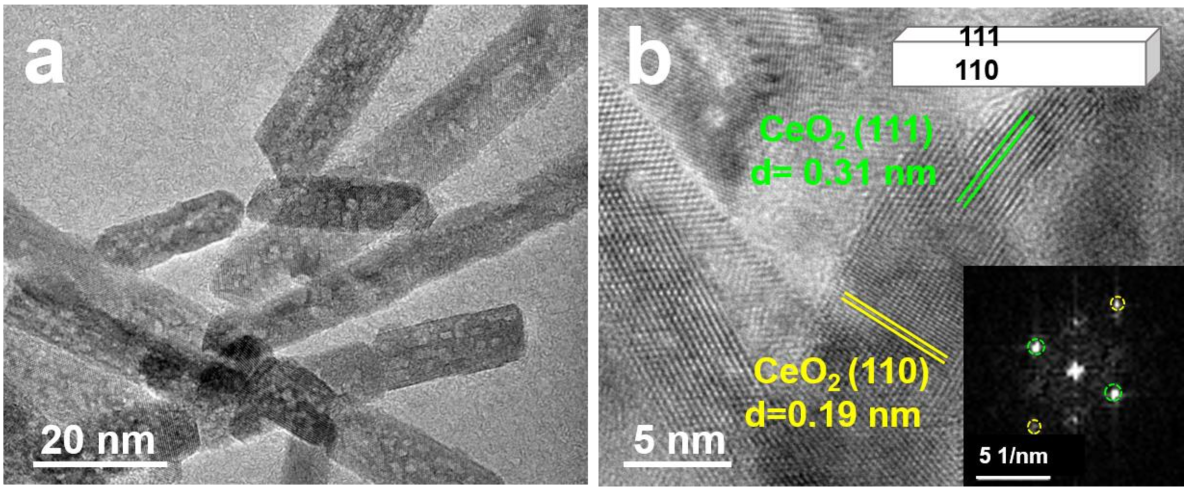

Supplementary Figure 3. a TEM and **b** local magnification HR-TEM images of CeO_2 support. The inset shows the corresponding FFT image.

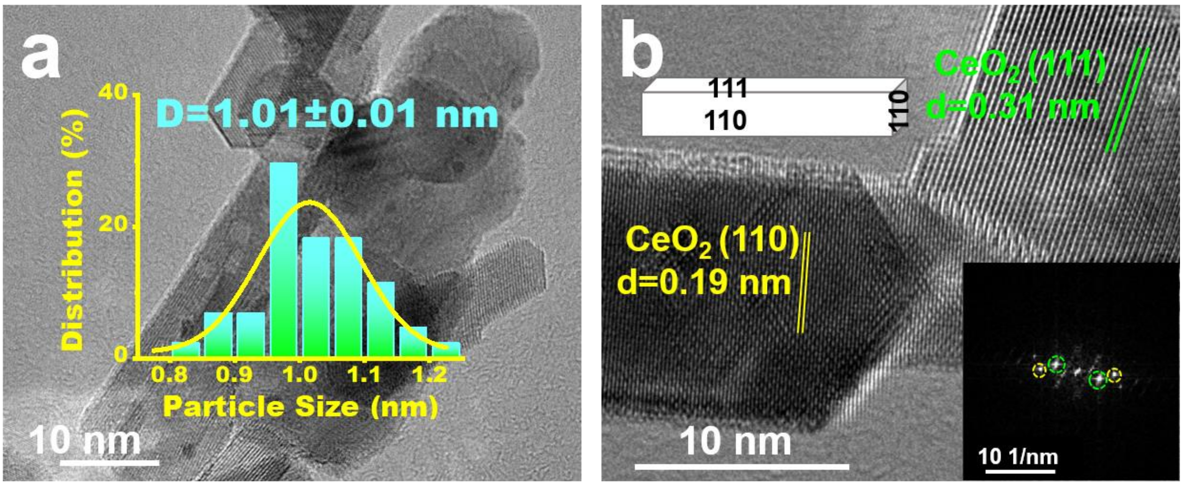

Supplementary Figure 4. **a** TEM and **b** local magnification HR-TEM images of 0.2% Ir/CeO_{2-x} sample. The insets show the histogram of Ir particle size distribution and corresponding FFT images, respectively.

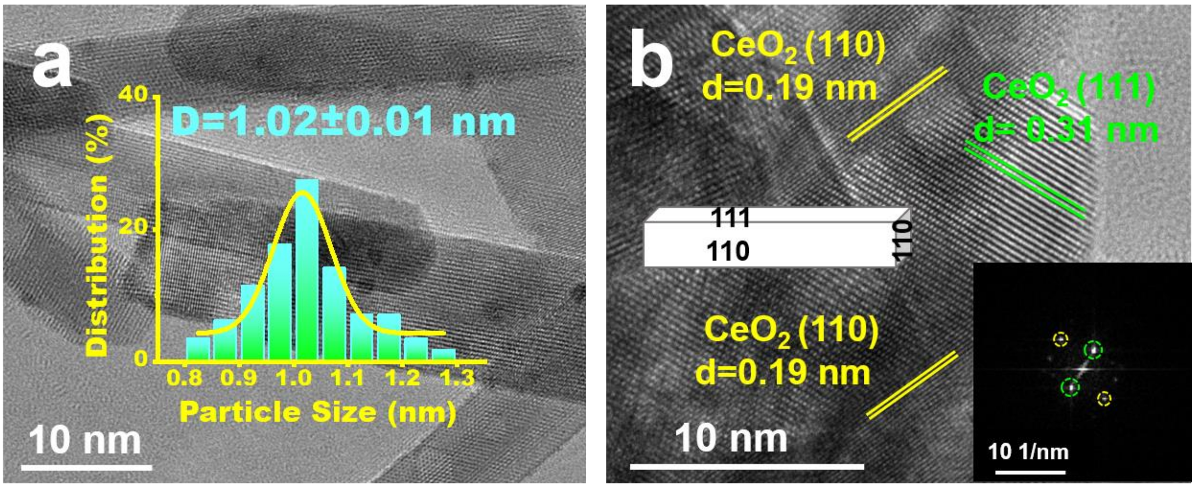

Supplementary Figure 5. **a** TEM and **b** local magnification HR-TEM images of 0.6% Ir/CeO_{2-x} sample. The insets show the histogram of Ir particle size distribution and corresponding FFT images, respectively.

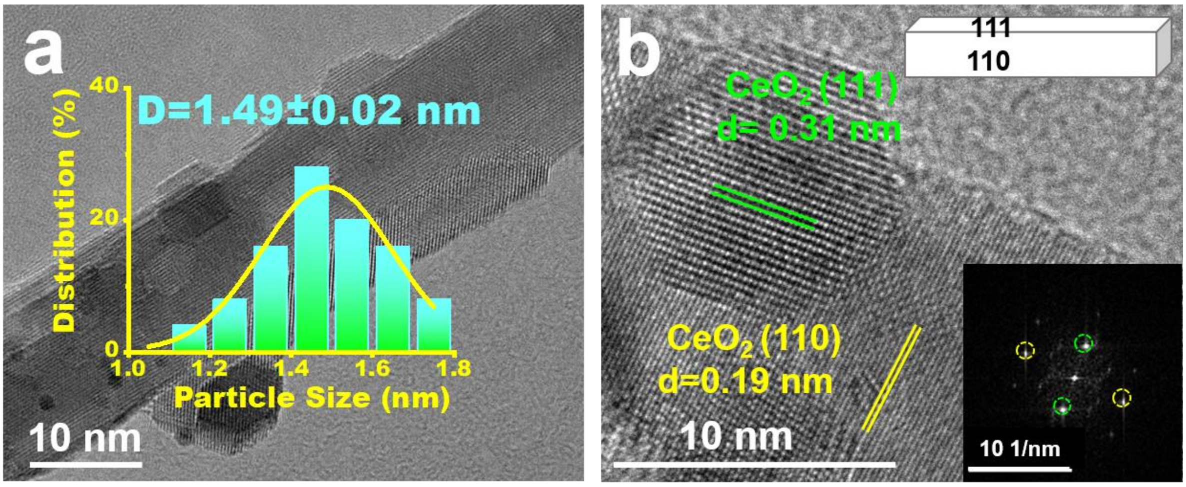

Supplementary Figure 6. a TEM and **b** local magnification HR-TEM images of 2.0% Ir/CeO_{2-x} sample. The insets show the histogram of Ir particle size distribution and corresponding FFT images, respectively.

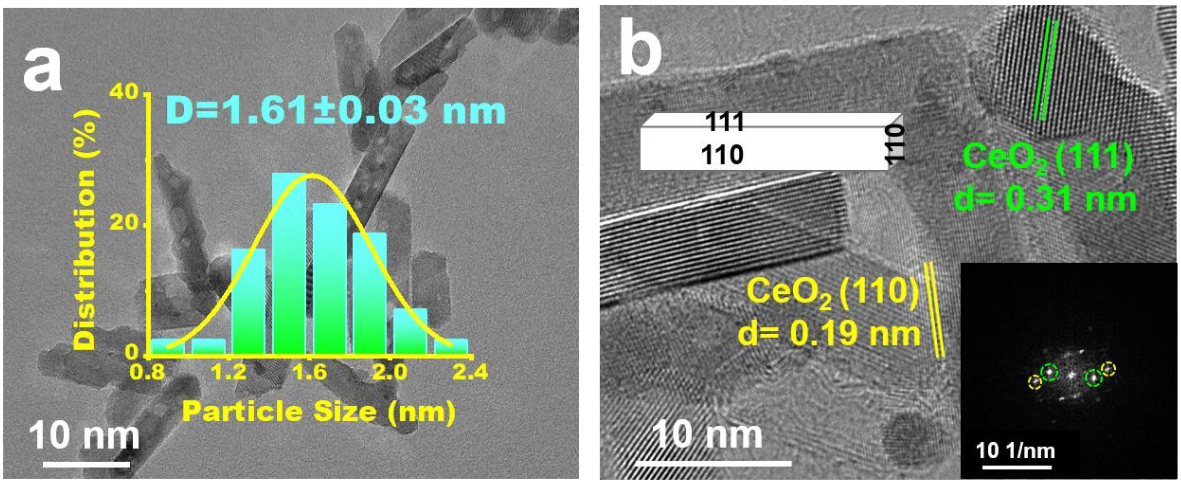

Supplementary Figure 7. **a** TEM and **b** local magnification HR-TEM images of 3.0% Ir/CeO_{2-x} sample. The insets show the histogram of Ir particle size distribution and corresponding FFT images, respectively.

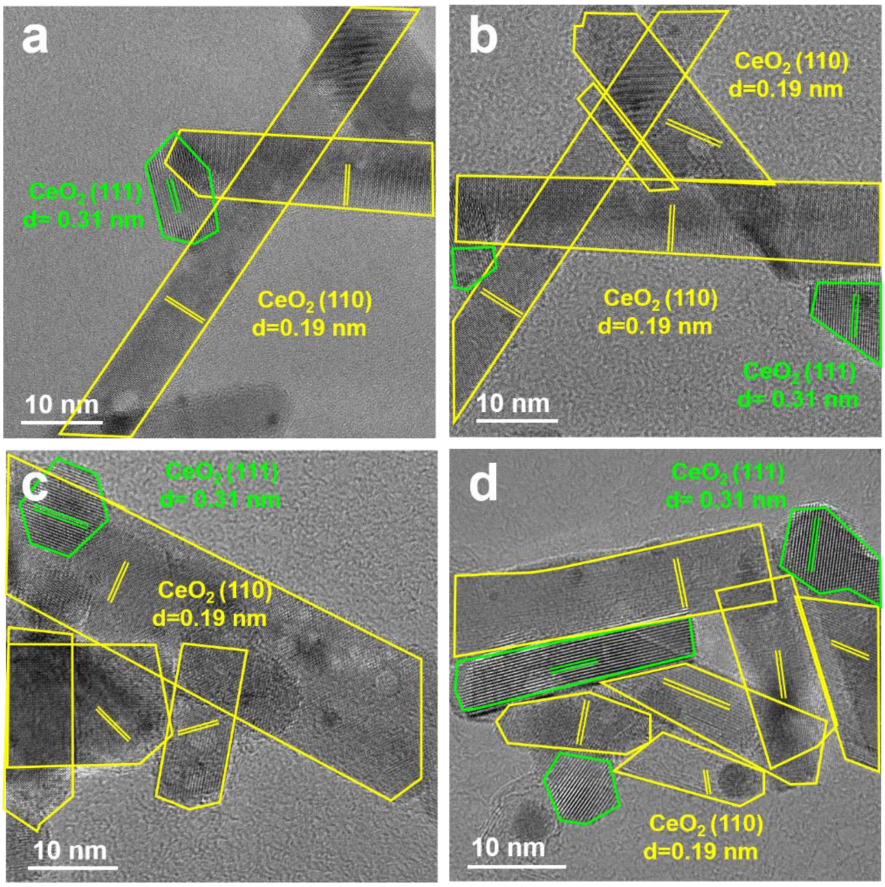

Supplementary Figure 8. a−d HR-TEM images of various Ir/CeO_{2-x} samples with Ir loading of 0.2%, 0.6%, 2.0% and 3.0%, respectively. The yellow and green borders represent the exposed (110) and (111) crystal facets of CeO_2 nanorods, respectively.

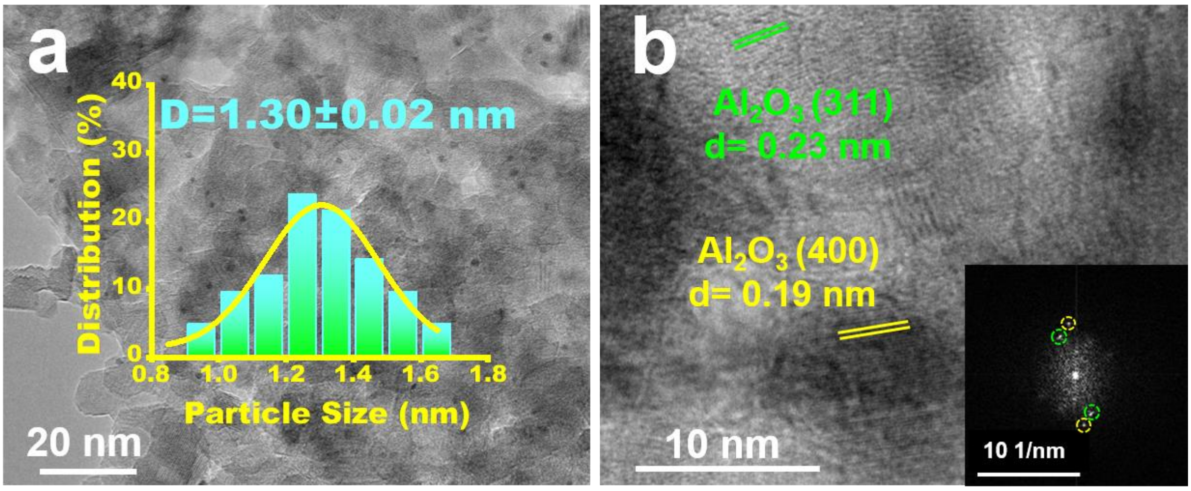

Supplementary Figure 9. a TEM and **b** HR-TEM images of 0.6% Ir/Al_2O_3 sample. The insets show the histogram of Ir particle size distribution and corresponding FFT images, respectively.

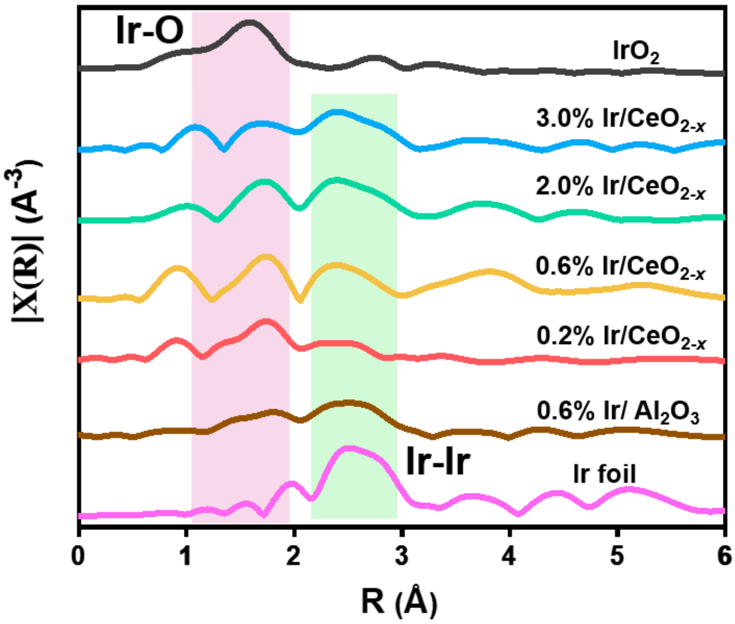

Supplementary Figure 10. EXAFS Fourier-transform spectra in R space for various samples.

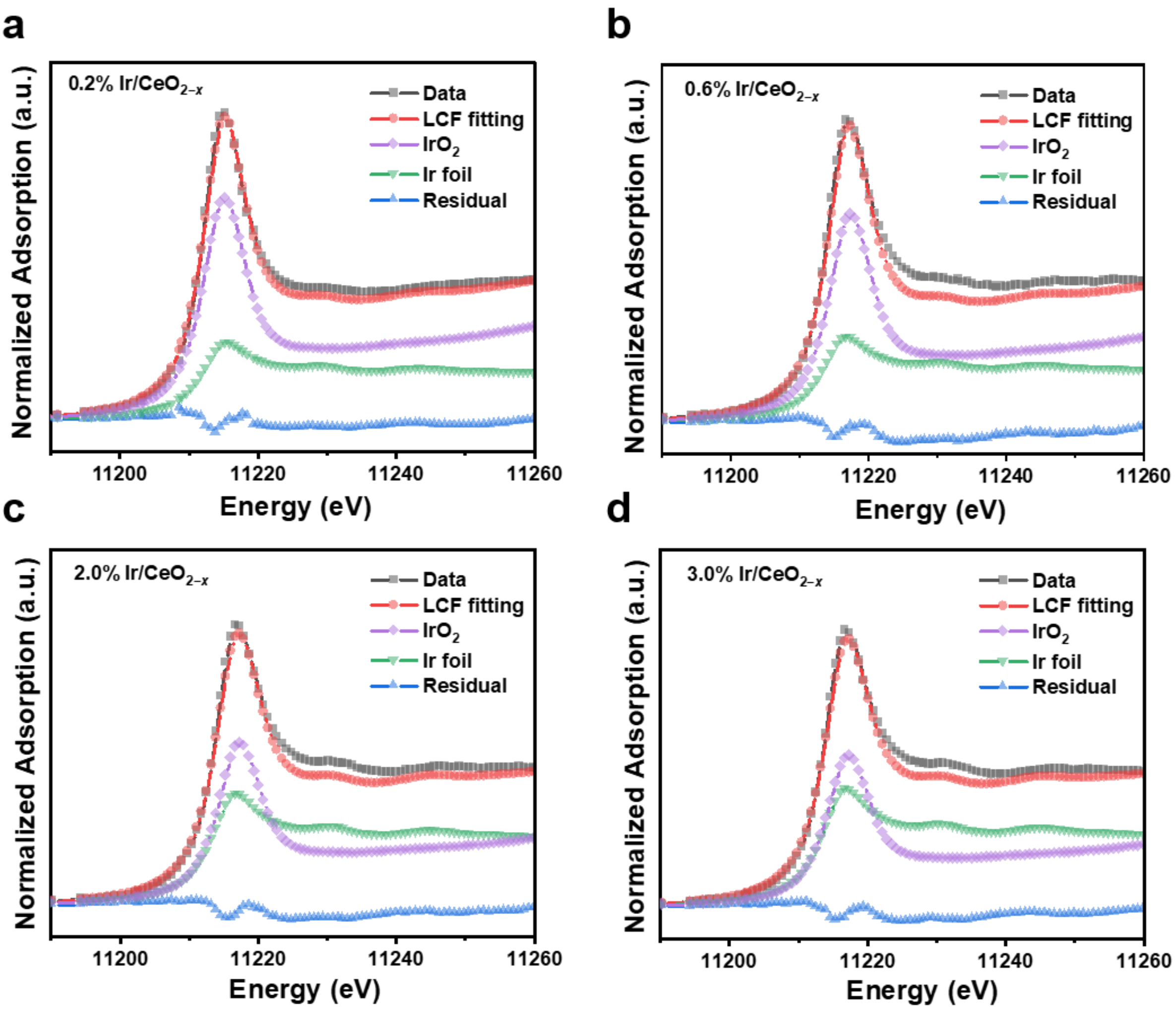

Supplementary Figure 11. a−d Linear combination fitting (LCF) curves of 0.2% Ir/CeO_{2-x}, 0.6% Ir/CeO_{2-x}, 2.0% Ir/CeO_{2-x}, and 3.0% Ir/CeO_{2-x} samples, respectively.

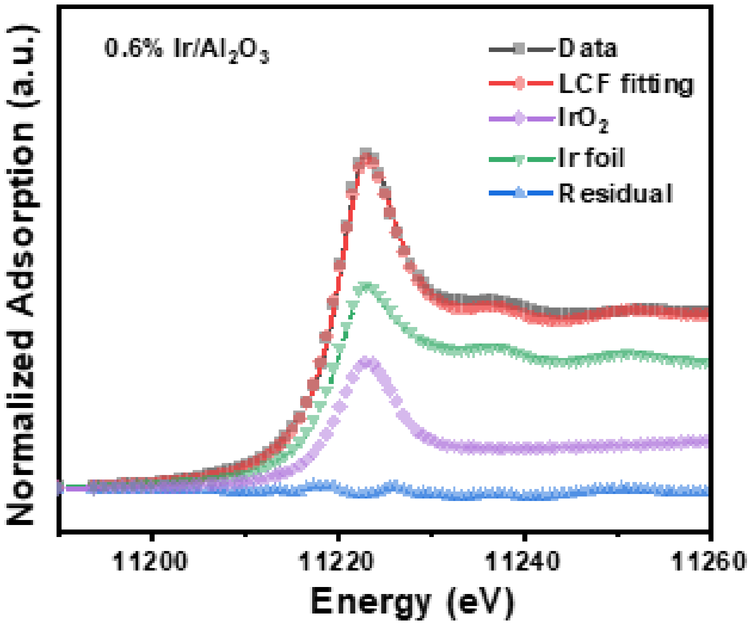

Supplementary Figure 12. Linear combination fitting (LCF) curve of 0.6% Ir/Al_2O_3 sample.

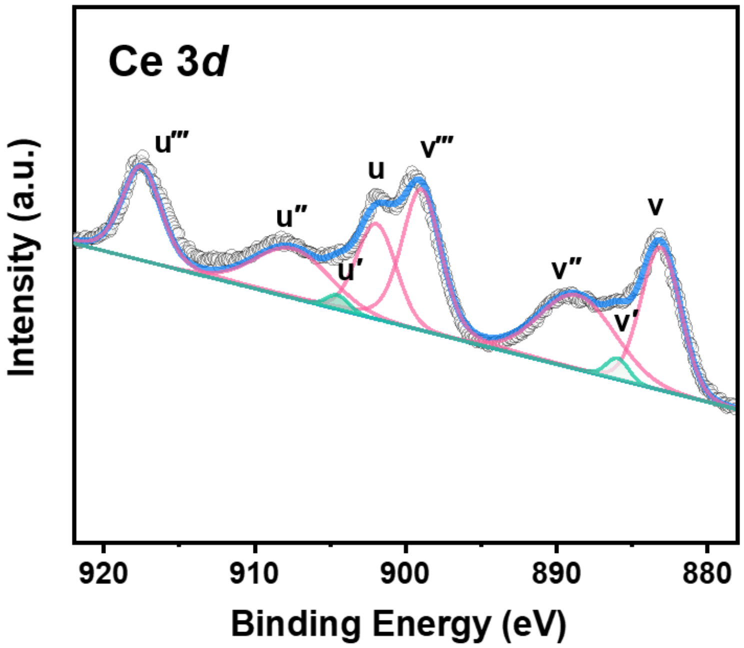

Supplementary Figure 13. *Quasi in situ* XPS of Ce 3*d* for CeO_2 nanorods support.

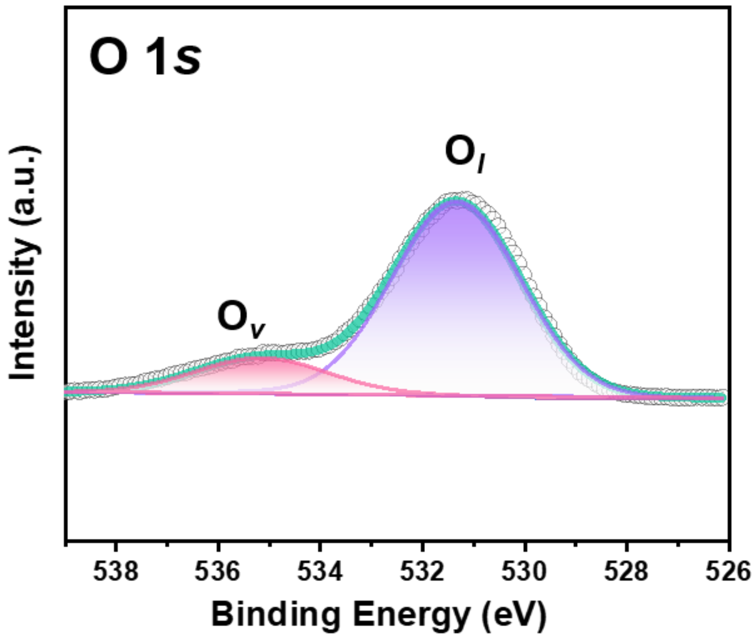

Supplementary Figure 14. *Quasi in situ* XPS of O 1*s* for CeO_2 nanorods support.

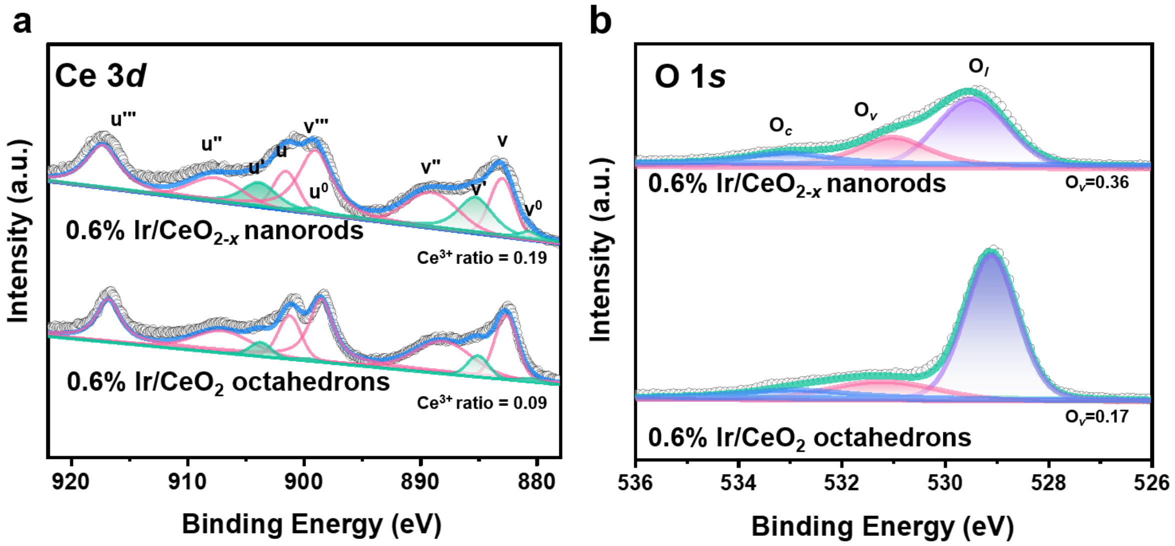

Supplementary Figure 15. *Quasi in situ* XPS of **a** Ce 3*d* and **b** O 1*s* for the 0.6% Ir/CeO_{2-x} nanorods and 0.6% Ir/CeO_{2-x} octahedrons samples, respectively.

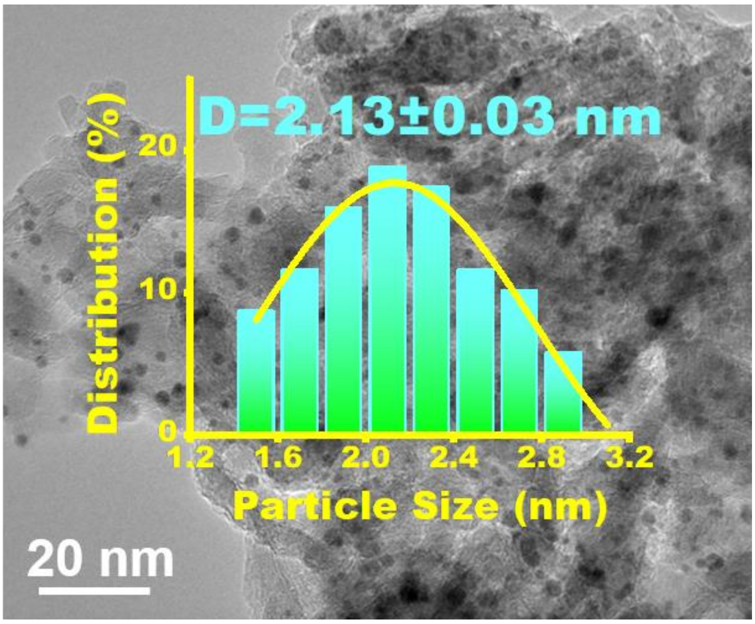

Supplementary Figure 16. TEM image of 0.6% Ir/Al_2O_3 sample after stability test for DRM at 700 °C. The inset shows the histogram of Ir particle size distribution.

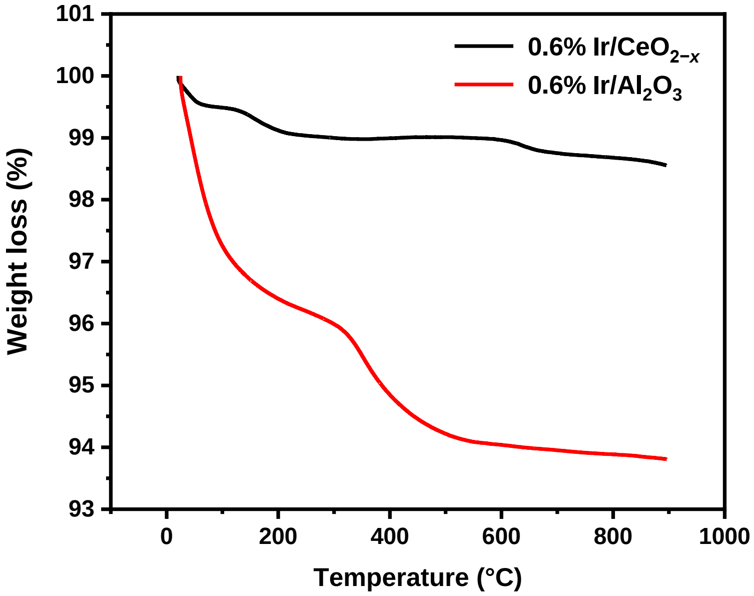

Supplementary Figure 17. TGA profiles of the used 0.6% Ir/CeO_{2-x} and 0.6% Ir/Al_2O_3 catalysts after stability test at 700 °C for DRM.

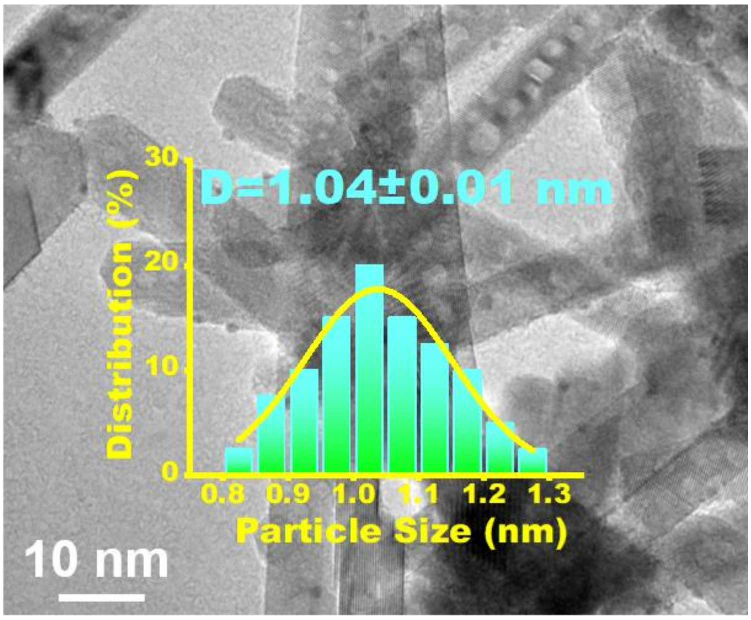

Supplementary Figure 18. TEM image of the used 0.6% Ir/CeO_{2-x} sample after stability test at 700 °C for DRM. The inset shows the histogram of Ir particle size distribution.

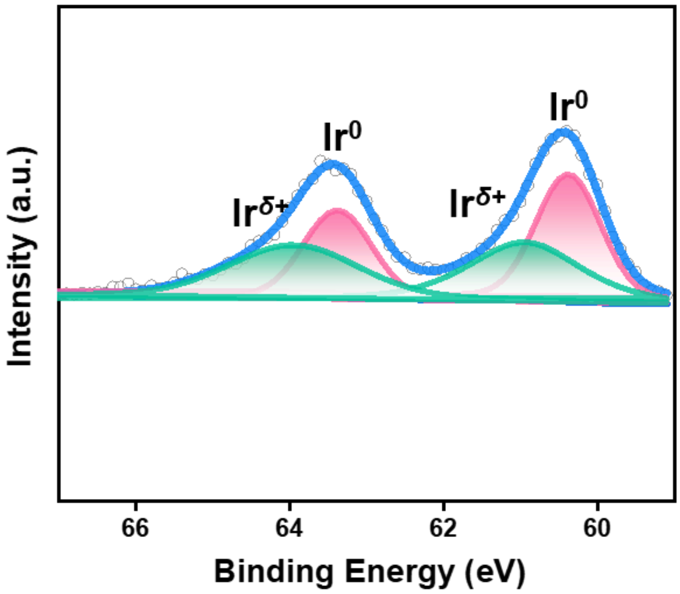

Supplementary Figure 19. *Quasi in situ* XPS of Ir 4*f* for the used 0.6% Ir/CeO_{2-x} sample after stability test at 700 °C for DRM.

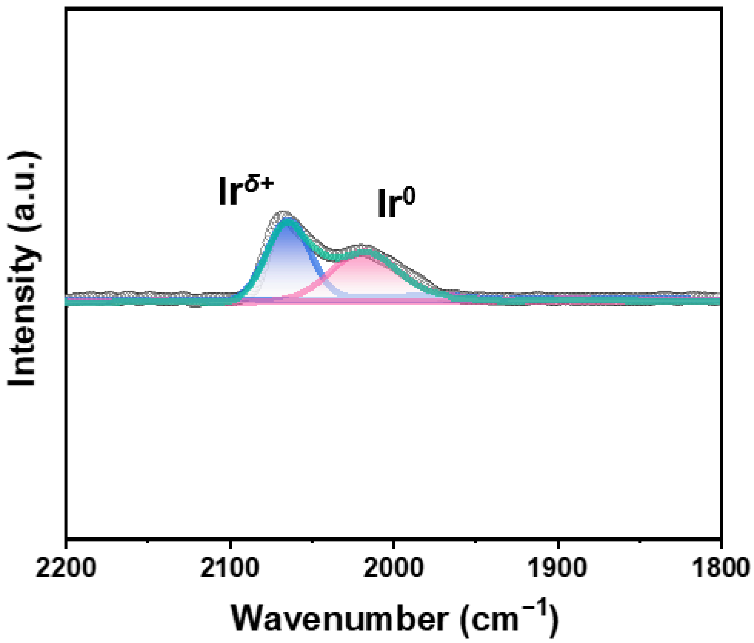

Supplementary Figure 20. CO-DRIFTS spectrum of the used 0.6% Ir/CeO_{2-x} sample after stability test at 700 °C for DRM.

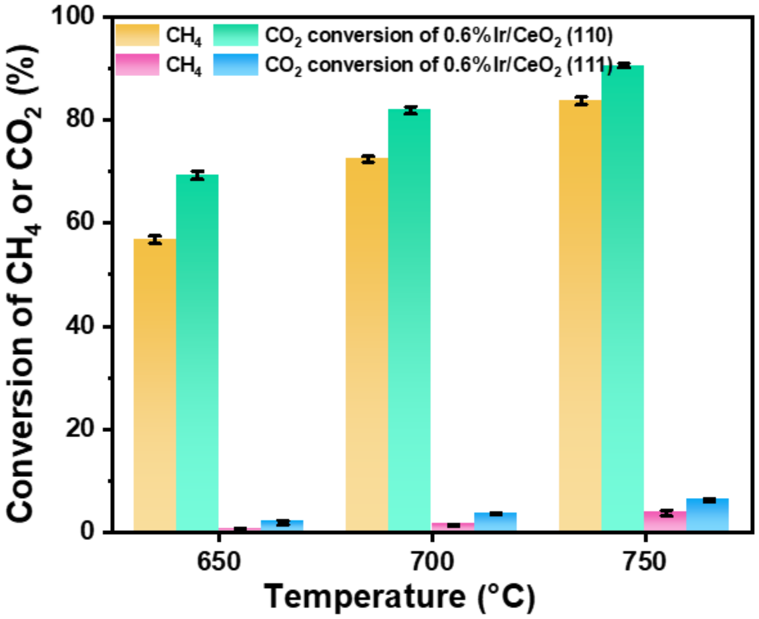

Supplementary Figure 21. CH_4 conversion and CO_2 conversion over the 0.6% Ir/CeO_{2-x} nanorods and 0.6% Ir/CeO_{2-x} octahedrons samples at 650, 700 and 750 °C, respectively.

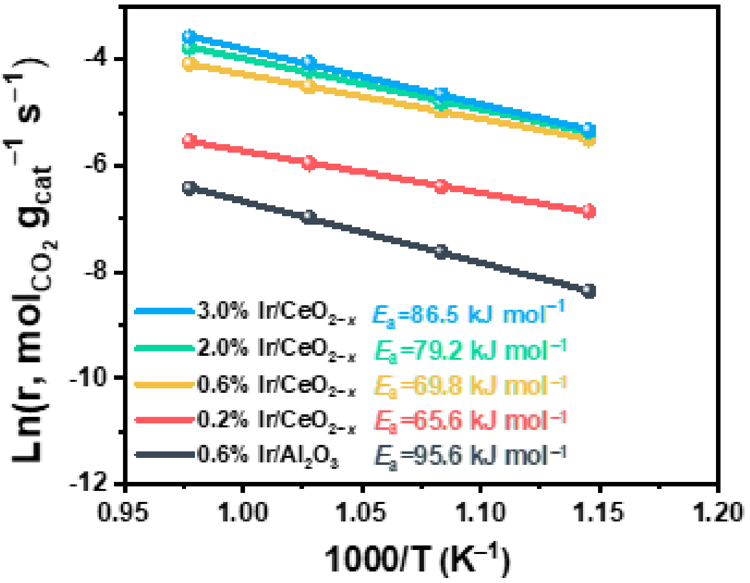

Supplementary Figure 22. Kinetic studies and activation energy (E_a) of CO_2 in DRM reaction over various catalysts.

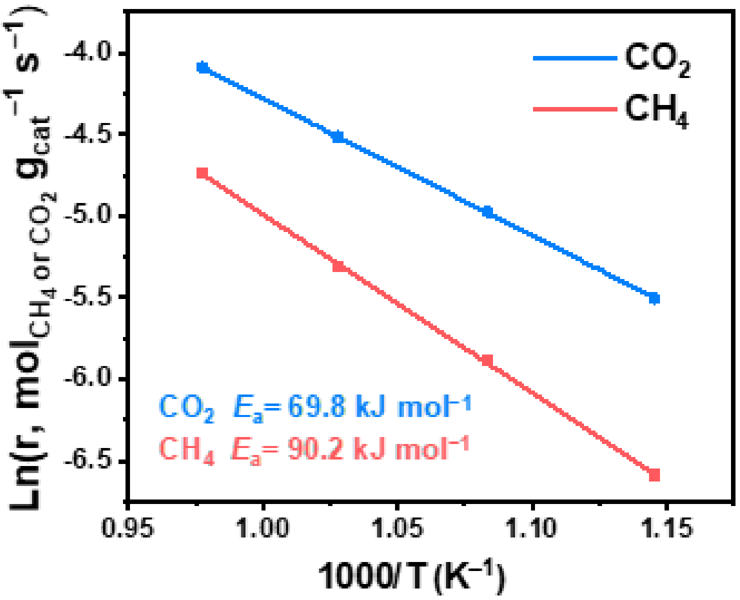

Supplementary Figure 23. Kinetic studies and activation energies (E_a) of CH_4 or CO_2 in DRM reaction over 0.6% Ir/CeO_{2-x}.

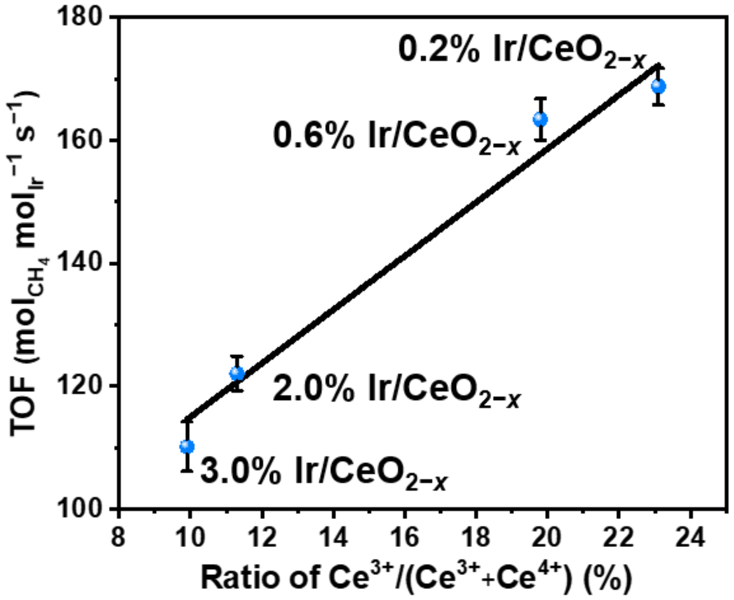

Supplementary Figure 24. TOF value as a function of surface concentration of $Ce^{3+}/(Ce^{3+} + Ce^{4+})$ ratio calculated by *quasi in situ* XPS results.

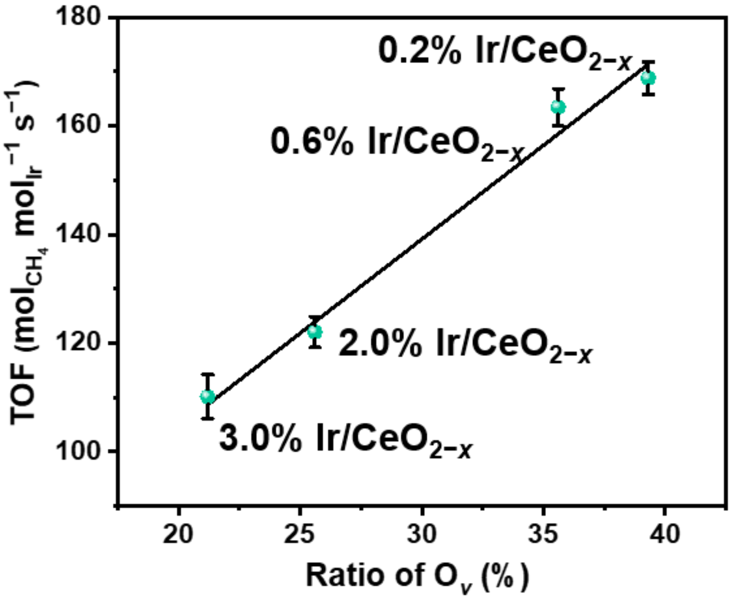

Supplementary Figure 25. TOF value as a function of surface oxygen vacancy ratio calculated by *quasi in situ* XPS results.

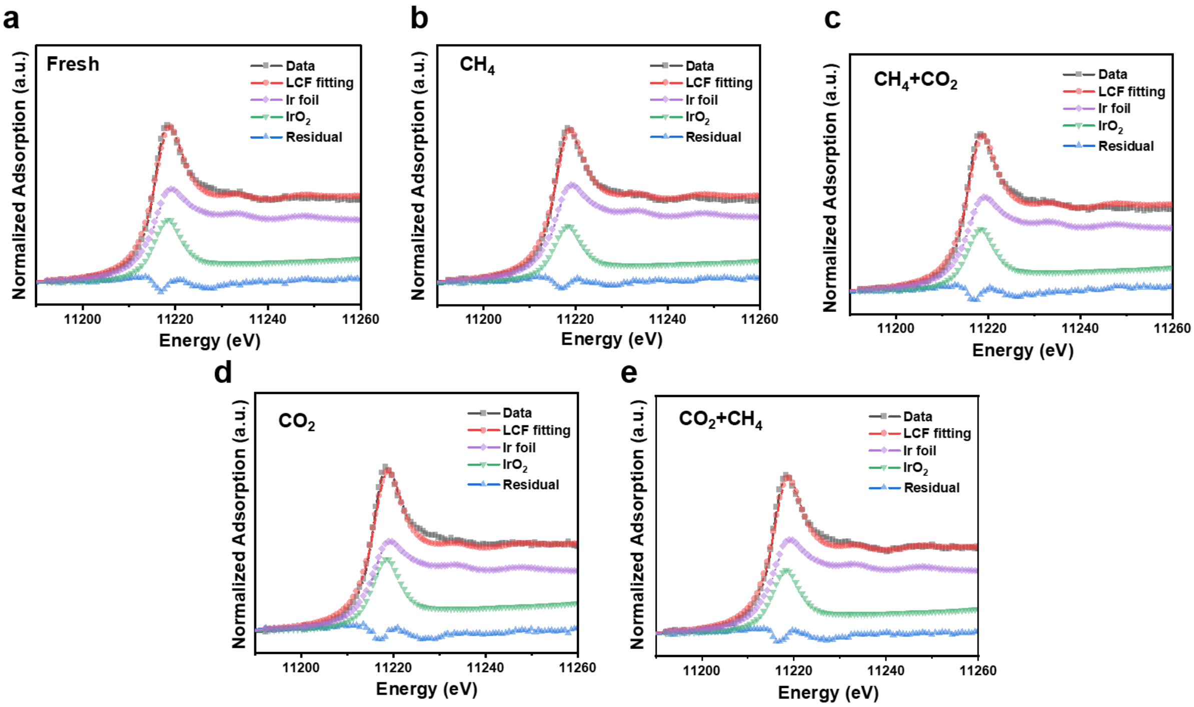

Supplementary Figure 26. Linear combination fitting (LCF) curves of **a** fresh 0.6% Ir/CeO_{2-x} at Ir L_3-edge with **b** CH_4, **c** CH_4+CO_2 treatment, **d** CO_2, and **e** CO_2+CH_4 treatment, respectively.

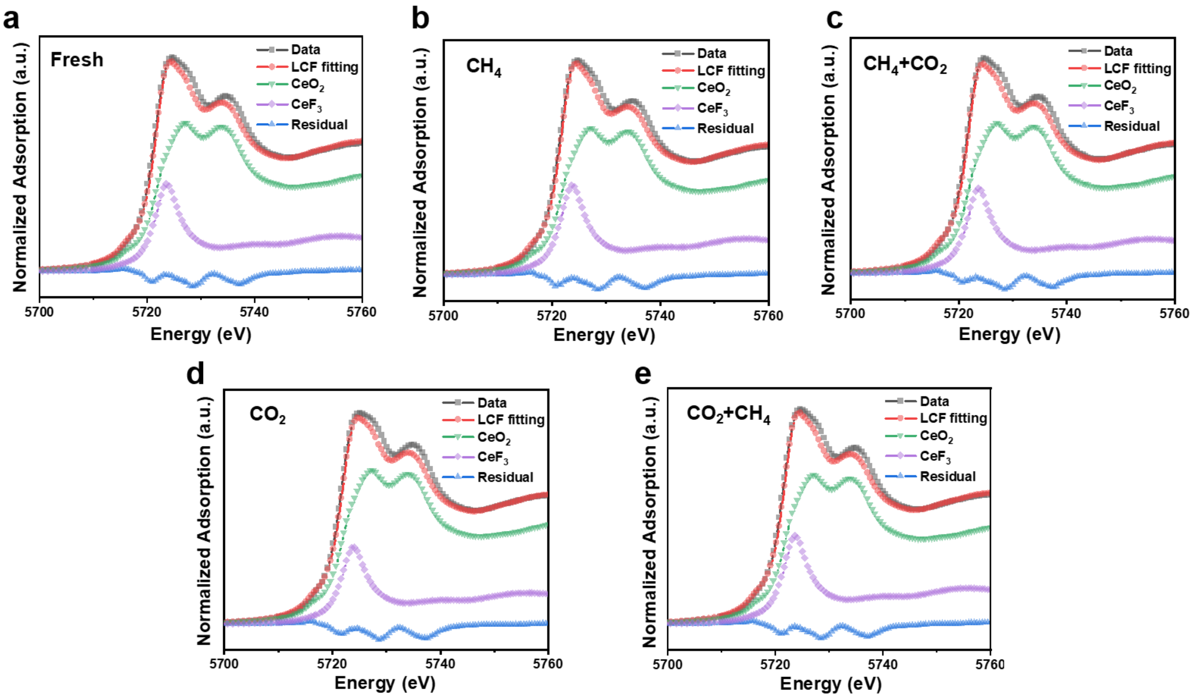

Supplementary Figure 27. Linear combination fitting (LCF) curves of **a** fresh 0.6% Ir/CeO_{2-x} at Ce L_3-edge with **b** CH_4, **c** CH_4+CO_2 treatment, **d** CO_2, and **e** CO_2+CH_4 treatment, respectively.

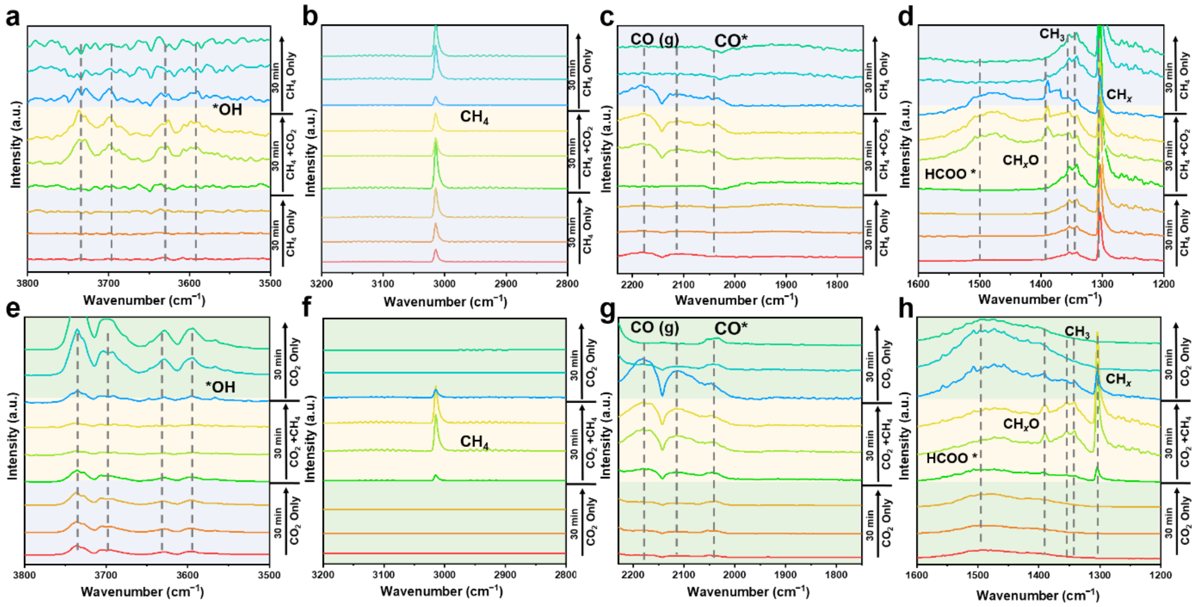

Supplementary Figure 28. *In situ/operando* DRIFTS spectra over 0.6% Ir/CeO_{2-x} sample at 700 °C after *in-situ* pretreatment and He purging, followed by exposure to: **a−d** first CH_4 atmosphere, subsequent CH_4+CO_2 and then CH_4 atmosphere for 30 min, respectively; **e−h** first CO_2 atmosphere, subsequent CO_2+CH_4 and then CO_2 atmosphere for 30 min, respectively.

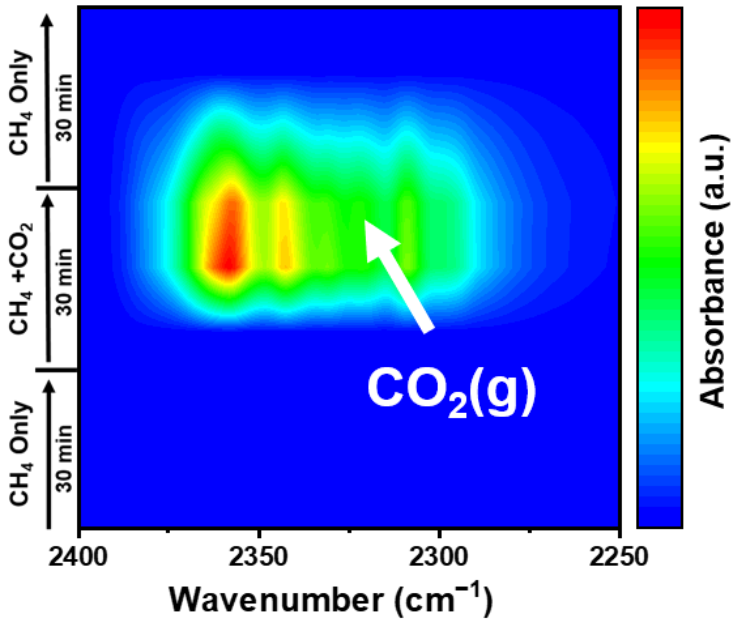

Supplementary Figure 29. *In situ/operando* DRIFTS spectra in the presence of 0.6% Ir/CeO_{2-x} at 700 °C after *in situ* pretreatment and He purging, followed by exposure to: first CH_4 atmosphere subsequent CH_4+CO_2 and finally CH_4 atmosphere for 30 min, respectively.

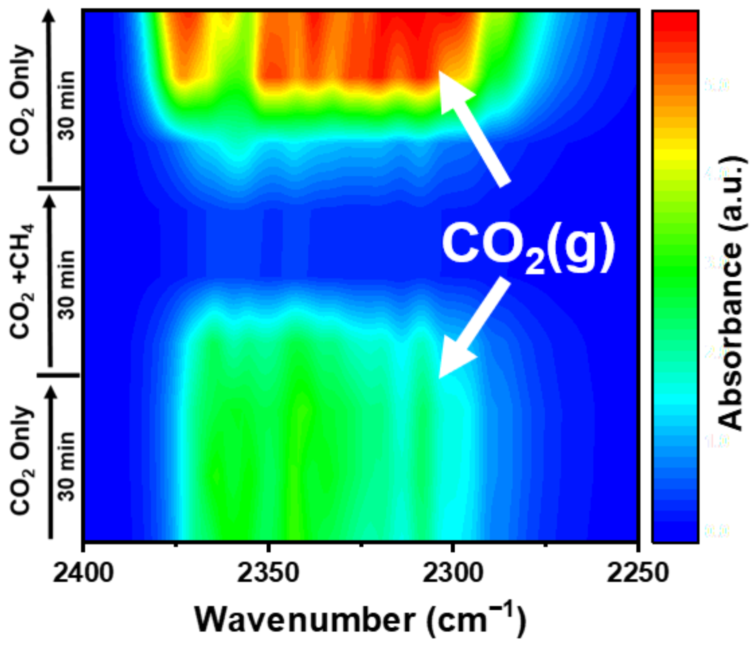

Supplementary Figure 30. *In situ/operando* DRIFTS spectra in the presence of 0.6% Ir/CeO_{2-x} at 700 °C after *in situ* pretreatment and He purging, followed by exposure to: first CO_2 atmosphere, subsequent CH_4+CO_2 and finally CO_2 atmosphere for 30 min, respectively.

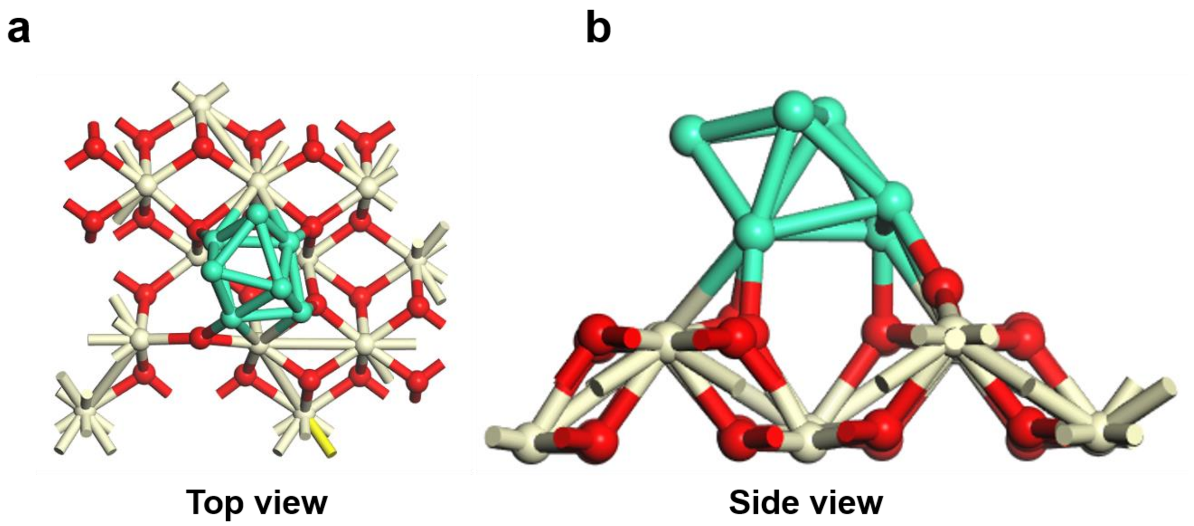

Supplementary Figure 31. a Top view and **b** side view of the Ir_7/CeO_{2-x} model for DFT calculations. Ir, green; Ce, yellow; O, crimson.

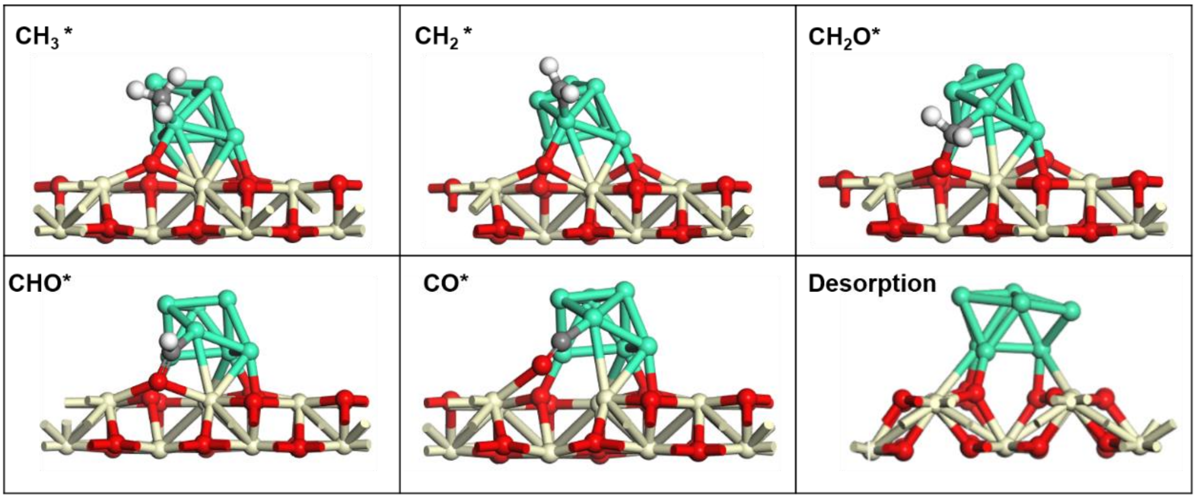

Supplementary Figure 32. Schematic illustration for the CH_4 decomposition at the interface of Ir/CeO_{2-x}. Ir, green; Ce, yellow; C, grey; O, crimson; H, white.

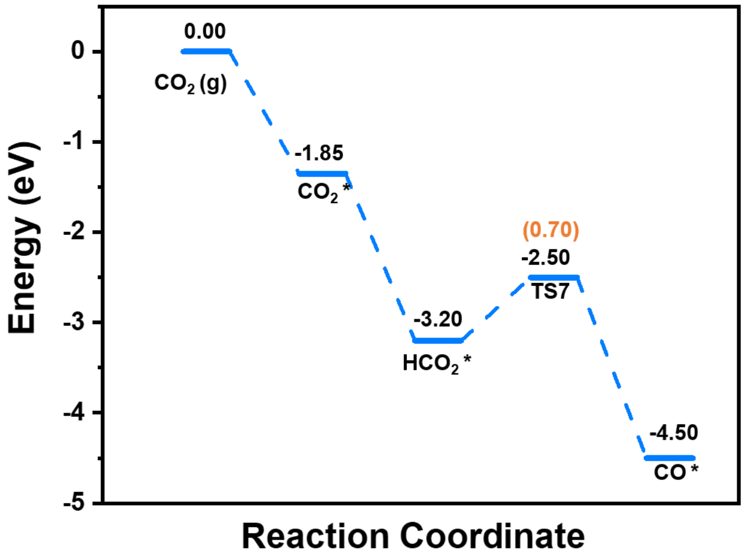

Supplementary Figure 33. The potential energy profiles for CO_2 decomposition on Ir/CeO_{2-x} (110) based on DFT calculations. 'TS' denotes a transition state. The black and orange numbers represent the adsorption energies and energy barriers of the elementary steps, respectively.

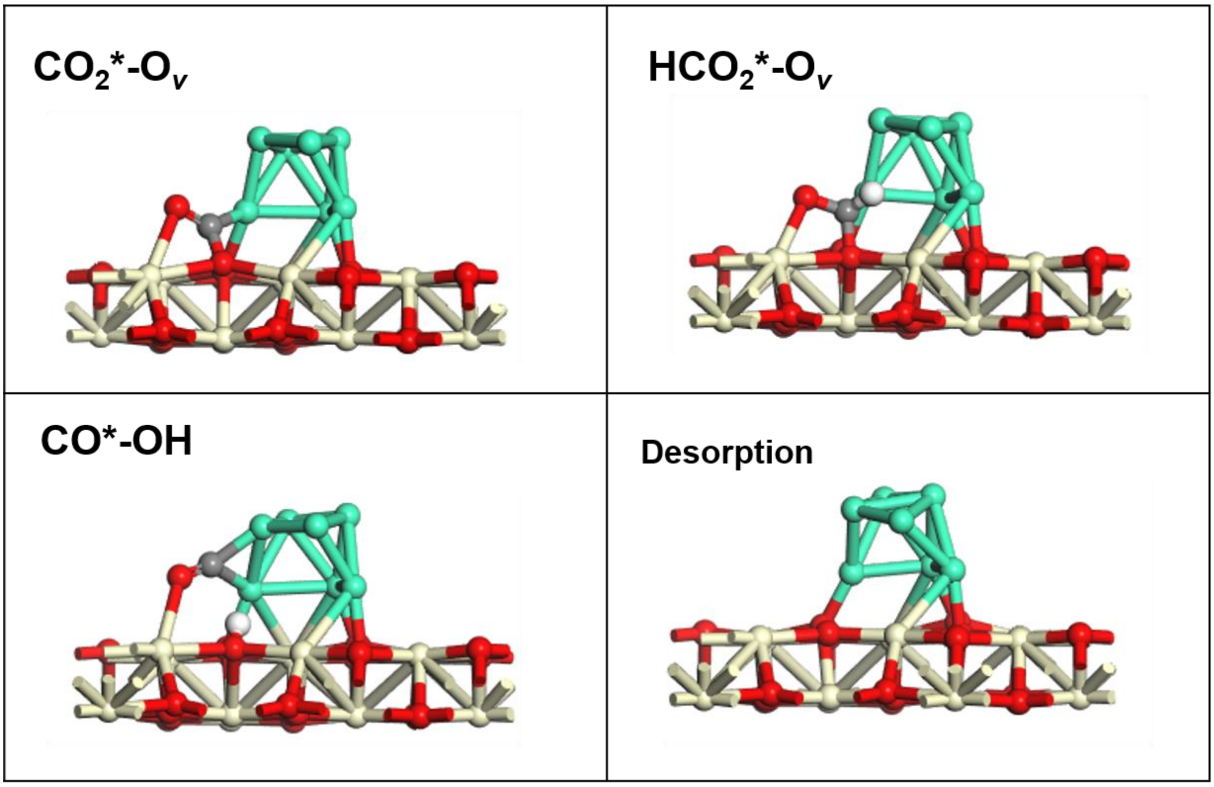

Supplementary Figure 34. Schematic illustration for the CO_2 decomposition at the interface of Ir/CeO_{2-x}. Ir, green; Ce, yellow; C, grey; O, crimson; H, white.

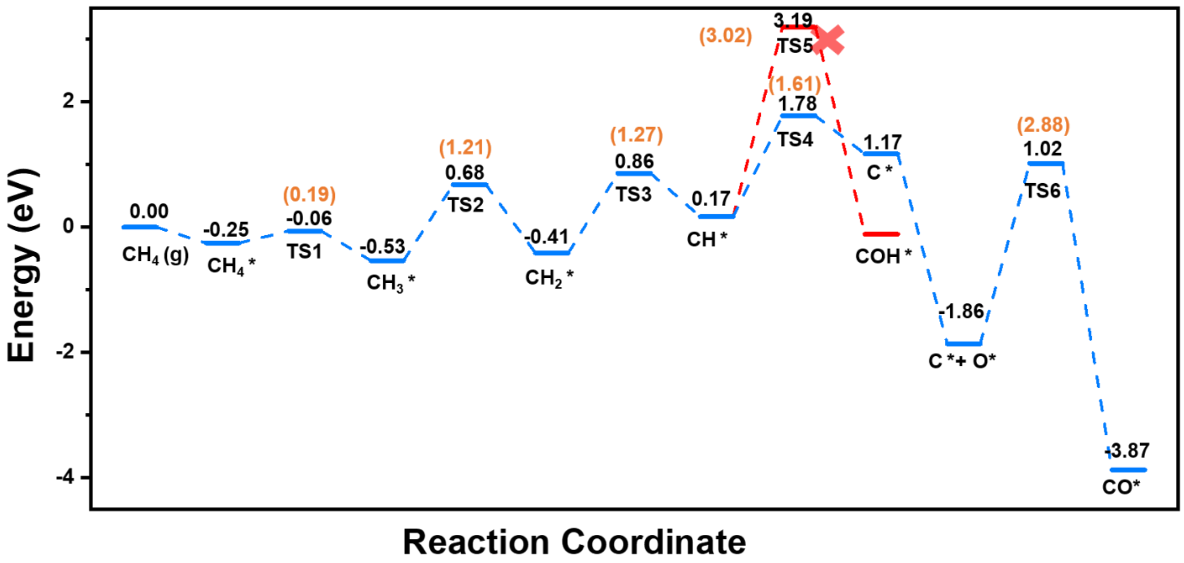

Supplementary Figure 35. The potential energy profiles for CH_4 decomposition on Ir/Al_2O_3 based on DFT calculations. 'TS' denotes a transition state. The black and orange numbers represent the adsorption energies and energy barriers of the elementary steps, respectively.

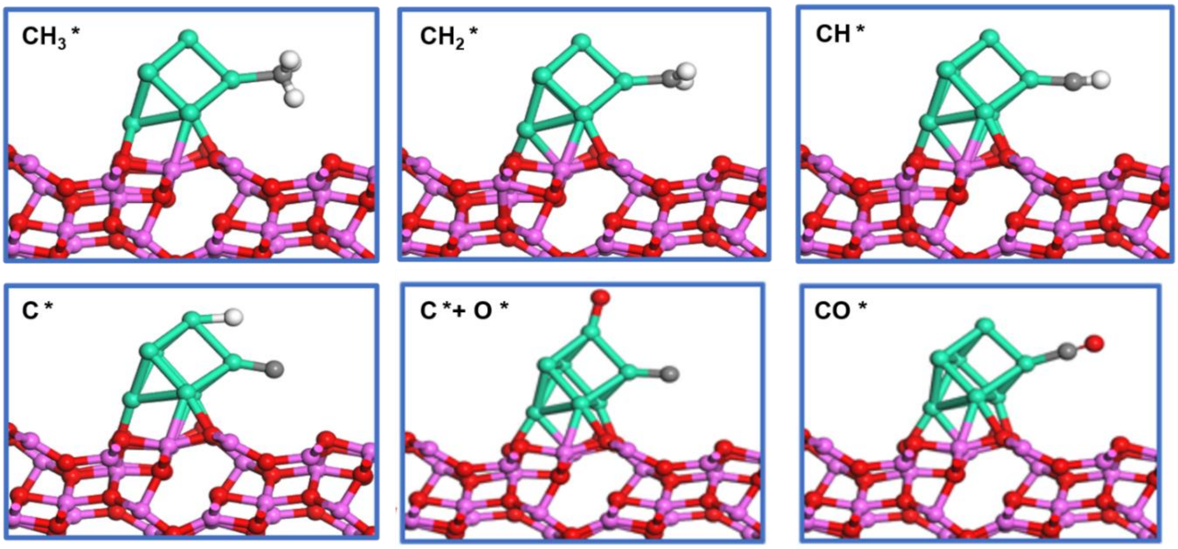

Supplementary Figure 36. Schematic illustration for the CH_4 decomposition on Ir/Al_2O_3. Ir, green; Al, pink; C, grey; O, red; H, white.

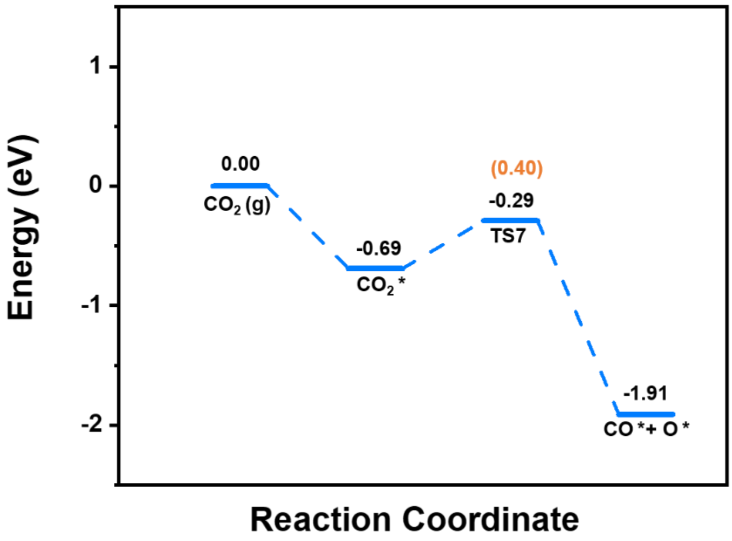

Supplementary Figure 37. The potential energy profiles for CO_2 decomposition on Ir/Al_2O_3 based on DFT calculations. 'TS' denotes a transition state. The black and orange numbers represent the adsorption energies and energy barriers of the elementary steps, respectively.

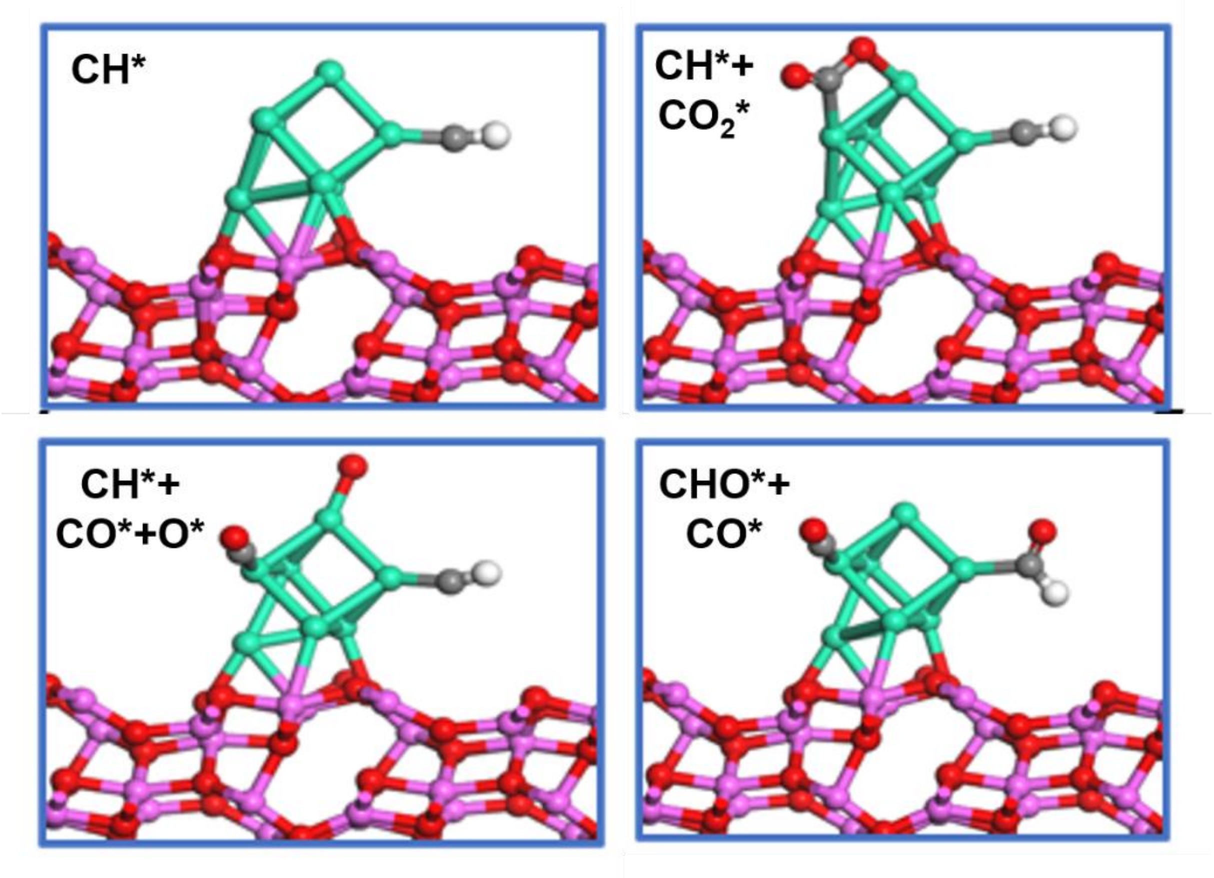

Supplementary Figure 38. Schematic illustration for the CO_2 decomposition on Ir/Al_2O_3. Ir, green; Al, pink; C, grey; O, red; H, white.

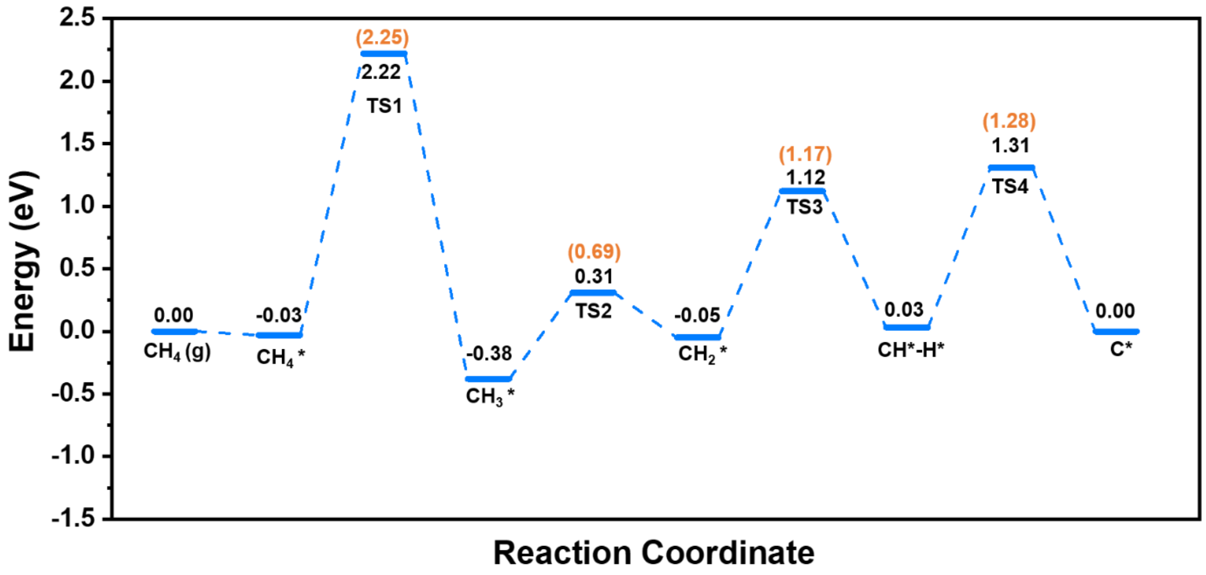

Supplementary Figure 39. Potential energy profile for CH_4 decomposition on the surface of Ir/CeO_2 (111) based on DFT calculations. 'TS' denotes a transition state. The black and orange numbers represent the adsorption energy and energy barrier of elementary steps, respectively.

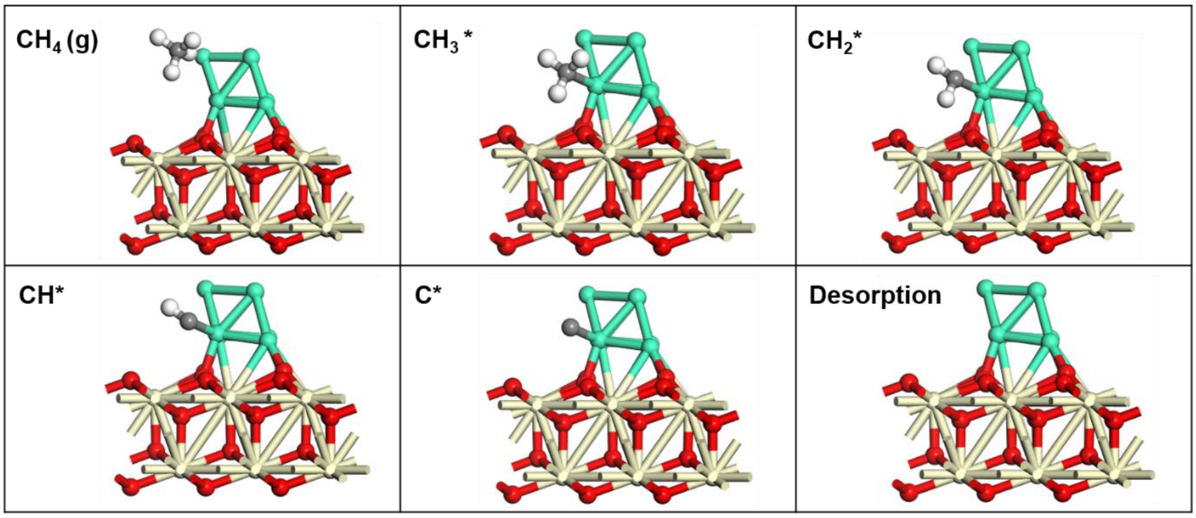

Supplementary Figure 40. Schematic illustration for the CH_4 decomposition on Ir/CeO_2 (111). Ir, green; Ce, yellow; C, grey; O, crimson; H, white.

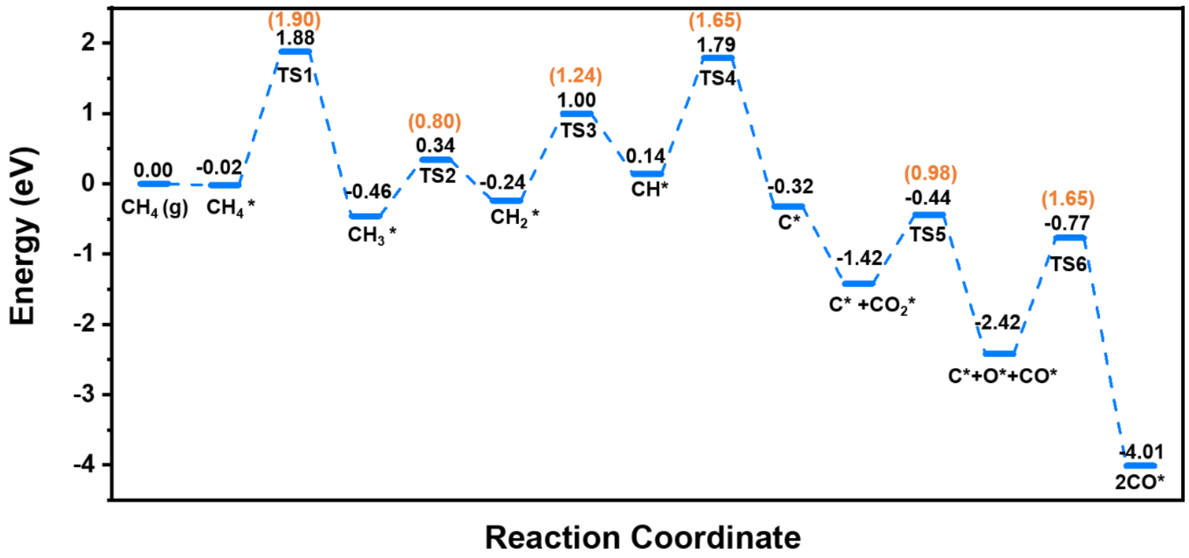

Supplementary Figure 41. Potential energy profile for CH4 and CO2 decomposition on Ir/CeO2 (110) without oxygen vacancy based on DFT calculations. 'TS' denotes a transition state. The black and orange numbers represent the adsorption energy and energy barrier of elementary steps, respectively.

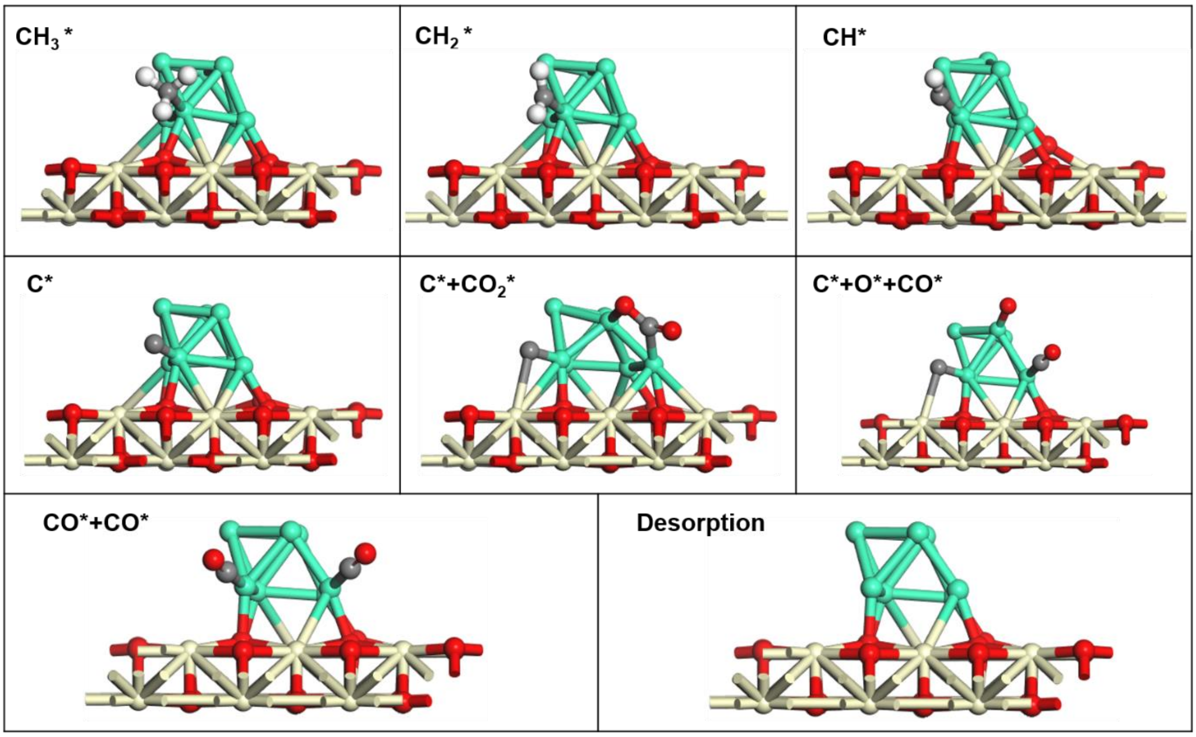

Supplementary Figure 42. Schematic illustration for CH_4 and CO_2 decomposition on Ir/CeO_2 (110) without oxygen vacancy. Ir, green; Ce, yellow; C, grey; O, crimson; H, white.

Supplementary Table 1. Physicochemical properties of various samples

Sample	Ir content[a] (*wt.*%)	BET Surface Area[b] ($m^2 \cdot g^{-1}$)	Mean pore size[b] (nm)	Mean Ir particle size[c] (nm)	Theoretical dispersion of Ir[d] (%)	Determined dispersion of Ir[e] (%)
0.6%Ir/Al_2O_3	0.6	/	/	1.3	76.9	65.2
0.2% Ir/CeO_{2-x}	0.2	91.9	22.6	1.0	99.0	81.9
0.6% Ir/CeO_{2-x}	0.6	87.9	22.4	1.0	98.0	79.1
2.0% Ir/CeO_{2-x}	2.0	85.2	21.2	1.5	66.7	45.7
3.0% Ir/CeO_{2-x}	3.0	83.6	20.4	1.6	62.5	38.9

[a] Ir loading was calculated based on ICP-AES results.

[b] Specific surface area and mean pore size were determined by BET measurements.

[c] The mean Ir size was determined by TEM images.

[d] Theoretical dispersion of Ir was estimated on the basis of Ir particle size from TEM (assuming Ir as a spherical particle).

[e] Determined dispersion of Ir was obtained from CO pulse chemisorption experiments results.

Supplementary Table 2. Relative concentration of surface species derived from deconvoluted XPS results

Sample	$Ir^{\delta+}/(Ir^{\delta+}+Ir^{0})$ ratio	$Ce^{3+}/(Ce^{3+}+Ce^{4+})$ ratio	Surface oxygen vacancy ratio
0.6%Ir/Al_2O_3	0%	--	--
0.2% Ir/CeO_{2-x}	57.3%	23.1%	39.3%
0.6% Ir/CeO_{2-x}	53.9%	19.8%	35.6%
2.0% Ir/CeO_{2-x}	34.1%	11.3%	25.6%
3.0% Ir/CeO_{2-x}	30.0%	9.9%	21.2%

Supplementary Table 3. Relative concentration of $Ir^{\delta+}$/ ($Ir^{\delta+}$+Ir^0) derived from *in situ* CO-DRIFTS

Sample	Linearly adsorbed CO		Gem-dicarbonyl species		$Ir^{\delta+}/(Ir^{\delta+}+Ir^0)$ Ratio
	Wavenumber (cm^{-1})	Area	Wavenumber (cm^{-1})	Area	
0.6%Ir/Al_2O_3	2017	1.71	--	--	--
0.2% Ir/CeO_{2-x}	2015	0.19	2062	0.24	56.0%
0.6% Ir/CeO_{2-x}	2020	0.91	2062	1.01	52.6%
2.0% Ir/CeO_{2-x}	2021	1.48	2061	0.66	30.7%
3.0% Ir/CeO_{2-x}	2021	6.49	2060	2.01	23.5%

Supplementary Table 4. LCF fitting results of the Ir L_3-edge for various samples

Sample	Ir^{4+} atomic ratio	Ir^{0} atomic ratio	Average valence state of Ir species
0.6%Ir/Al_2O_3	26.9%	73.1%	+1.1
0.2% Ir/CeO_{2-x}	63.2%	36.8%	+2.5
0.6% Ir/CeO_{2-x}	59.2%	40.8%	+2.4
2.0% Ir/CeO_{2-x}	46.2%	53.8%	+1.8
3.0% Ir/CeO_{2-x}	43.0%	57.0%	+1.7

Supplementary Table 5. Comparison studies on catalytic performance of various catalysts for DRM

Sample	Tem. (°C)	GHSV (mL g^{-1} h^{-1})	Con.CH_4 (%)	Con.CO_2 (%)	H_2/CO	Reaction rate (μmol$_{CH4}$ g_{cat}^{-1} s^{-1})	Ref.
**0.6% Ir/CeO_{2-x}**	**650**	**240000**	**~57**	**~69**	**0.82**	**763**	**This work**
	**700**	**240000**	**~72**	**~82**	**0.86**	**973**	
	**750**	**240000**	**~84**	**~91**	**0.89**	**1125**	
2% Ir/$Ce_{0.9}Pr_{0.1}O_2$-DP	750	18000	61	75	0.97	68	4
Ir/$Ce_{0.9}Pr_{0.1}O_2$	750	18000	57	76	--	64	5
Rh-$La_2Ti_2O_7$	700	10000	~77	71	--	58	6
Pd/MgO/Al_2O_3	700	24000	89	89	0.88	67	7
Pd5Y/Al_2O_3	700	12000	~89	94	~0.85	67	8
$Sm_2Ru_{0.2}Ce_{1.8}O_7$	700	288000	~30	~45	--	448	9
Ru/Mg_3(Al)O	750	30000	84	90	~0.92	157	10
0.8%Rh/CeO_2+ZrO_2	750	120000	~52	~68	~0.85	388	11
2.0 wt.% Rh/MCF	750	54000	~91	~94	~0.95	306	12
0.6%Ni-0.44%Ir/SiO_2	700	38400	~35	44	0.58	84	13
0.2Pd/5Ni-MgO	750	70000	97	96	0.99	60	14
$Ru_{0.035}Ni_{0.035}Mg_{0.93}O$-DR	760	86000	~83	~90	~0.83	442	15
Co-Ru-Zr-silica	800	32000	87	99	~0.9	173	16
NiAuPtAlCe	750	36000	88	94	--	99	17
Pt Co/CeO_2	600	12000	~32	--	~0.65	24	18
	700	12000	~73	--	~0.94	55	
Pt-Ni/Al_2O_3	700	12000	69	76	0.66	52	19
SiO_2@Ni@ZrO_2	700	16000	58	48	~0.85	58	20
Ni/CeZrO_2	700	120000	51	66	~0.85	380	21
Ni/h-BN	750	48000	73	80	--	218	22

Supplementary Table 6. Kinetic parameters used for fitting the experimental data in **Fig. 3e−f**

$R_{CH4} = k_1 \cdot (P_{CH4})^{a1} \cdot (P_{CO2})^{b1}$			$R_{CO2} = k_2 \cdot (P_{CH4})^{a2} \cdot (P_{CO2})^{b2}$		
k_1	**a1**	**a2**	k_2	**a1**	**a2**
0.021	0.67	0.09	0.029	0.53	0.07

Supplementary References

1. Meng, H. et al. A strong bimetal-support interaction in ethanol steam reforming. *Nat. Commun.* **14**, 3189 (2023).

2. Wang, J. et al. Design of a carbon-resistant Ni@S−2 reforming catalyst: controllable Ni nanoparticles sandwiched in a peasecod-like structure. *Appl. Catal. B Environ.* **282**, 119546 (2021).

3. Yan, X. et al. Highly efficient and stable Ni/CeO_2−SiO_2 catalyst for dry reforming of methane: effect of interfacial structure of Ni/CeO_2 on SiO_2. *Appl. Catal. B Environ.* **246**, 221−231 (2019).

4. Wang, F. et al. Tuning the metal-support interaction in catalysts for highly efficient methane dry reforming reaction. *Appl. Catal. B Environ.* **180**, 511−520 (2016).

5. Wang, F. et al. Enhanced catalytic performance of Ir catalysts supported on ceria-based solid solutions for methane dry reforming reaction. *Catal. Today* **281**, 295−303 (2017).

6. Wu, J. et al. Revealing the synergistic effects of Rh and substituted $La_2B_2O_7$ (B = Zr or Ti) for preserving the reactivity of catalyst in dry reforming of methane. *ACS Catal.* **9**, 932−945 (2018).

7. Shi, C. et al. Role of MgO over γ−Al_2O_3−supported Pd catalysts for carbon dioxide reforming of methane. *Appl. Catal. B Environ.* **170−171**, 43−52 (2015).

8. Shi, C. et al. Effect of a second metal (Y, K, Ca, Mn or Cu) addition on the carbon dioxide reforming of methane over nanostructured palladium catalysts. *Appl. Catal. B Environ.* **115−116**, 190−200 (2012).

9. Naeem, M. A. et al. Exsolution of metallic Ru nanoparticles from defective, fluorite-type solid solutions $Sm_2Ru_xCe_{2-x}O_7$ to impart stability on dry reforming catalysts. *ACS Catal.* **10**, 1923−1937 (2019).

10. Li, D. et al. Carbon dioxide reforming of methane over Ru catalysts supported on Mg−Al oxides: A highly dispersed and stable Ru/Mg(Al)O catalyst. *Appl. Catal. B Environ.* **200**, 566−577 (2017).

11. Yentekakis, I. V. et al. Effect of support oxygen storage capacity on the catalytic performance of Rh nanoparticles for CO2 reforming of methane. *Appl. Catal. B Environ.* **243**, 490−01 (2019).

12. Qian, L. et al. The promotion effect of hydrogen spillover on CH_4 reforming with CO_2 over Rh/MCF catalysts. *Appl. Catal. B Environ.* **164**, 168−175 (2015).

13. Wang, L. et al. Self-confinement created for a uniform Ir–Ni/SiO_2 catalyst with enhanced performances on CO_2 reforming of methane. *Energy Fuels* **34**, 111−117 (2019).

14. Singha, R. K. et al. Synthesis and catalytic activity of a Pd doped Ni–MgO catalyst for dry

reforming of methane. *J. Mater. Chem. A* **5**, 15688−15699 (2017).

15. Zhou, H. et al. A single source method to generate Ru−Ni−MgO catalysts for methane dry reforming and the kinetic effect of Ru on carbon deposition and gasification. *Appl. Catal. B Environ.* **233**, 143−159 (2018).

16. Whang, H. S. et al. Enhanced activity and durability of Ru catalyst dispersed on zirconia for dry reforming of methane. *Catal. Today* **293−294**, 122−128 (2017).

17. Wu, H. et al. Bi- and trimetallic Ni catalysts over Al_2O_3 and Al_2O_3−MO (M = Ce or Mg) oxides for methane dry reforming: Au and Pt additive effects. *Appl. Catal. B Environ.* **156−157**, 350−361 (2014).

18. Xie, Z. et al. Dry reforming of methane over CeO_2−supported Pt−Co catalysts with enhanced activity. *Appl. Catal. B Environ.* **236**, 280−293 (2018).

19. García-Diéguez, M. et al. Characterization of alumina-supported Pt, Ni and PtNi alloy catalysts for the dry reforming of methane. *J. Catal.* **274**, 11−20 (2010).

20. Dou, J. et al. Sandwiched SiO_2@Ni@ZrO_2 as a coke resistant nanocatalyst for dry reforming of methane. *Appl. Catal. B Environ.* **254**, 612−623 (2019).

21. Zhang, F. et al. Effects of Zr doping into ceria for the dry reforming of methane over Ni/$CeZrO_2$ catalysts: in situ studies with XRD, XAFS, and AP-XPS. *ACS Catal.* **10**, 3274−3284 (2020).

22. Dong, J. et al. Reaction-induced strong metal–support interactions between metals and inert boron nitride nanosheets. *J. Am. Chem. Soc.* **142**, 17167−17174 (2020).
